# Genomic loci and molecular genetic mechanisms for hidradenitis suppurativa

**DOI:** 10.1093/bjd/ljaf277

**Published:** 2025-07-12

**Authors:** K Alaine Broadaway, Quan Sun, Sharon N Edmiston, Kevin W Currin, Swarooparani Vadlamudi, Tyne W Miller-Fleming, Yue Shi, Kristen Fajgenbaum, Maria Melendez-Gonzalez, Helen Bui, Franklin R Blum, Linnea Westerkam, Rayad Shams, Teja Mallela, Brandt Levitt, Lan Lin, Honglin Hao, Aylin Memili, Peter Straub, Wei Zhou, Kathleen Mullan Harris, Alicia Martin, Nancy J Cox, Zhi Liu, Nancy E Thomas, Yun Li, Karen L Mohlke, Christopher J Sayed

**Affiliations:** Department of Genetics, University of North Carolina, Chapel Hill, NC, USA; Department of Biostatistics, University of North Carolina, Chapel Hill, NC, USA; Center for Computational and Genomic Medicine, Children’s Hospital of Philadelphia, Philadelphia, PA, USA; Department of Dermatology, University of North Carolina, Chapel Hill, NC, USA; Lineberger Comprehensive Cancer Center, University of North Carolina, Chapel Hill, NC, USA; Department of Genetics, University of North Carolina, Chapel Hill, NC, USA; Department of Genetics, University of North Carolina, Chapel Hill, NC, USA; Department of Medicine, Division of Genetic Medicine, Vanderbilt University Medical Center, Nashville, TN, USA; Vanderbilt Genetics Institute, Vanderbilt University Medical Center, Nashville, TN, USA; Program in Medical and Population Genetics, Broad Institute of MIT and Harvard, Cambridge, MA, USA; Stanley Center for Psychiatric Research, Broad Institute of MIT and Harvard, Cambridge, MA, USA; Analytic and Translational Genetics Unit, Massachusetts General Hospital, Boston, MA, USA; Nashville Skin Comprehensive Dermatology Center, Nashville, TN, USA; Department of Dermatology, University of North Carolina, Chapel Hill, NC, USA; Department of Dermatology, Howard University College of Medicine, Washington, DC, USA; Department of Dermatology, University of North Carolina, Chapel Hill, NC, USA; Department of Dermatology, University of North Carolina, Chapel Hill, NC, USA; School of Medicine, University of North Carolina, Chapel Hill, NC, USA; Department of Dermatology, University of North Carolina, Chapel Hill, NC, USA; Carolina Population Center, University of North Carolina, Chapel Hill, NC, USA; Department of Dermatology, University of North Carolina, Chapel Hill, NC, USA; Department of Dermatology, University of North Carolina, Chapel Hill, NC, USA; School of Medicine, University of North Carolina, Chapel Hill, NC, USA; Department of Medicine, Division of Genetic Medicine, Vanderbilt University Medical Center, Nashville, TN, USA; Vanderbilt Genetics Institute, Vanderbilt University Medical Center, Nashville, TN, USA; Program in Medical and Population Genetics, Broad Institute of MIT and Harvard, Cambridge, MA, USA; Stanley Center for Psychiatric Research, Broad Institute of MIT and Harvard, Cambridge, MA, USA; Psychiatric and Neurodevelopmental Genetics Unit, Center for Genomic Medicine, Massachusetts General Hospital, Boston, MA, USA; Carolina Population Center, University of North Carolina, Chapel Hill, NC, USA; Sociology Department, University of North Carolina at Chapel Hill, Chapel Hill, NC, USA; Program in Medical and Population Genetics, Broad Institute of MIT and Harvard, Cambridge, MA, USA; Stanley Center for Psychiatric Research, Broad Institute of MIT and Harvard, Cambridge, MA, USA; Analytic and Translational Genetics Unit, Massachusetts General Hospital, Boston, MA, USA; Department of Medicine, Division of Genetic Medicine, Vanderbilt University Medical Center, Nashville, TN, USA; Vanderbilt Genetics Institute, Vanderbilt University Medical Center, Nashville, TN, USA; Department of Dermatology, University of North Carolina, Chapel Hill, NC, USA; Lineberger Comprehensive Cancer Center, University of North Carolina, Chapel Hill, NC, USA; Department of Dermatology, University of North Carolina, Chapel Hill, NC, USA; Lineberger Comprehensive Cancer Center, University of North Carolina, Chapel Hill, NC, USA; Department of Genetics, University of North Carolina, Chapel Hill, NC, USA; Department of Biostatistics, University of North Carolina, Chapel Hill, NC, USA; Department of Genetics, University of North Carolina, Chapel Hill, NC, USA; Department of Dermatology, University of North Carolina, Chapel Hill, NC, USA

## Abstract

**Background:**

Hidradenitis suppurativa (HS) is a common, chronic and debilitating inflammatory disease that most commonly affects intertriginous skin. Despite its high heritability, the genetic underpinnings of HS remain poorly understood.

**Objectives:**

To identify genetic signals associated with HS, determine genetic relationships with other diseases and investigate potential molecular genetic mechanisms.

**Methods:**

We performed a genome-wide association meta-analysis of six studies, totalling 4540 patients with HS and > 1 million control participants, and identified genetic correlations with other common diseases. We integrated the HS data with expression quantitative trait loci from 10 trait-relevant tissues, epigenomic and transcriptomic data from human scalp, differential expression data from HS lesions vs. adjacent skin and mesenchymal Hi-C chromatin looping data. To identify functional noncoding variants, we performed transcriptional reporter assays for signals near *KLF5* and *SOX9*.

**Results:**

We identified 11 significant HS signals across 7 loci: 4 corresponded to previously reported associations, 4 represented novel signals within known loci and 3 were signals in newly implicated loci. We identified significant genetic correlations between HS and other inflammatory conditions, particularly inflammatory bowel disease, rheumatoid arthritis, type 2 diabetes mellitus and asthma. We prioritized candidate genes for the 11 signals. The risk allele at *KLF5* exhibited 10-fold greater transcriptional activity than the nonrisk allele, while risk alleles at *SOX9* showed significantly reduced transcriptional activity.

**Conclusions:**

Our results provide insights into potential genetic mechanisms underlying HS and suggest potential therapeutic targets for this challenging condition.

## Teaser

We performed a genome-wide meta-analysis of hidradenitis suppurativa (HS), identifying 11 significant risk signals – 7 of them novel. Integration with functional genomics highlighted candidate genes and mechanisms, including noncoding variants that modulate KLF5 and SOX9 activity. These findings offer insight into the biology of HS and suggest new directions for therapeutic development.

## Graphical Abstract

**Graphical Abstract:**
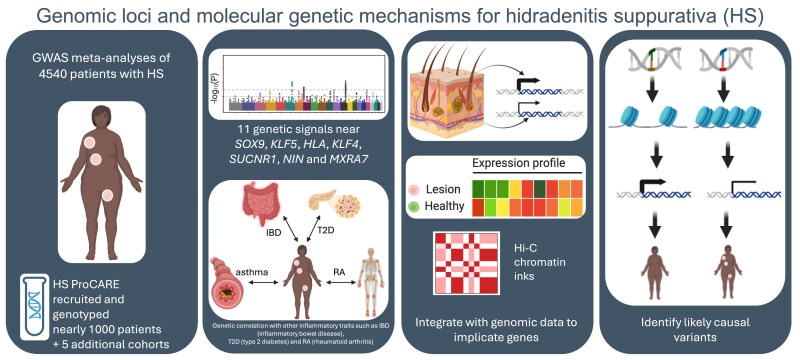

Linked Article: Simmonds and Khan *Br J Dermatol* 2025; **193**:816–817.

What is already known about this topic?

- Hidradenitis suppurativa (HS) is a chronic inflammatory skin disease with a strong genetic component.
- Prior common variant genome-wide association studies have identified six genetic loci associated with HS, but the underlying biological mechanisms remain poorly understood.

What does this study add?

- We identified 11 genetic signals, including 7 novel ones.
- Integrative analyses found genetic links between HS and other inflammatory diseases.
- Our study prioritized potential causal genes and functional follow-up identified variants near *SOX9* and *KLF5* that likely drive two noncoding signals.

What is the translational message?

- Our findings reveal biological pathways involving keratinocyte function and inflammation, suggesting potential therapeutic targets for HS.
- Identifying causal genes could inform precision medicine approaches for treatment.

Hidradenitis suppurativa (HS) is a highly heritable chronic inflammatory skin disease characterized by recurrent painful nodules, abscesses and tunnels, primarily affecting intertriginous regions.^1–4^ HS is associated with many comorbidities, including psoriasis, inflammatory bowel disease (IBD) and polycystic ovary syndrome (PCOS).^5–7^ Owing to the complexity of HS pathophysiology, the manifestations and progressions of HS phenotypes are highly heterogeneous, as observed by differences across environmental and genetic factors.^8,9^

Genetic studies have begun to shed light on the aetiology of HS. Rare variants analyses have pointed to genes related to the γ-secretase complex and autoinflammatory pathways.^3,10–14^ Two genome-wide association studies (GWAS) have identified common variants associated with HS. Our previous meta-analysis of HS Program for Research and Care Excellence (ProCARE) and two biobanks identified associations near *SOX9* and *KLF5*.^15^ More recently, a meta-analysis of Nordic, UK and US-based studies provided further support for these signals and identified four additional common-variant signals: one in *WNT10A*; one near *TMED10*; and intergenic signals on chromosomes 6 and 9.^14^

Despite these discoveries, much of HS heritability remains unexplained, and the functional consequences of GWAS associations across populations are unclear. To address these gaps, we expanded ProCARE and conducted a GWAS meta-analysis across 6 studies with 4540 patients, identifying 11 association signals. To link these signals to potential genes, we integrated them with transcriptomic and epigenomic data. Lastly, to investigate plausible causal regulatory variants, we analysed allelic differences in transcriptional activity of variants at *KLF5* and *SOX9*, providing mechanistic evidence of variant effects.

## Materials and methods

### Genetic association and meta-analyses

We expanded the HS ProCARE study to 986 patients and used METAL to conduct a GWAS meta-analysis across six studies (Table S1; see Supporting Information).^16^ To control for population structure, we stratified analyses by population: self-reported Black/African participants (AFR) and all other participants [European and admixed American (EUR + AMR); Table S1]. A GWAS was performed separately in each cohort by population group before meta-analysis. We first performed a GWAS meta-analysis by combining all population groups from all cohorts. Next, we performed analyses stratified by population group, separating AFR participants from all other participants, and then performed analyses stratified by sex (Table S2; see Supporting Information).

We defined a locus as a lead variant with *P*-value < 5 × 10^–8^ and all variants within 1 Mb of the lead variant, extending to 2 Mb in the HLA region due to extensive linkage disequilibrium (LD). A locus can harbour several genetic signals distinctly associated with HS; to identify conditionally distinct signals within a locus, we performed approximate conditional analysis using genome-wide complex trait analysis (Appendix S1; see Supporting Information).^17,18^

### Fine-mapping

To identify likely causal variants at each locus, we used the Bayesian fine-mapping method SuSiE (susieR package 0.12.35) to identify 95% credible sets (Appendix S1).^19^ To match a signal to a credible set, we required that the lead variant be in the credible set.

### Genetic correlation with comorbid traits

We applied LD score regression to estimate the genetic correlation between HS and coeliac disease, IBD, PCOS, rheumatoid arthritis (RA), type 2 diabetes (T2D), atopic dermatitis, psoriasis, schizophrenia and asthma (Appendix S1).^20–30^ We reviewed the lead HS variants in the GWAS Catalog,^31^ to identify reported associations with related traits.

### Candidate genes and regulatory elements

To prioritize potential candidate genes, we generated variant-level annotation using the WGSA Annotator,^32^ tested for co-localization between HS signals and signals from 10 sources of expression quantitative trait loci (eQTL) data in HS-relevant tissues,^33–37^ and we queried if genes were differentially expressed between cell types of the scalp or differentially expressed in HS lesions vs. unaffected skin from patients with HS (Appendix S1).^38,39^

To investigate potential regulatory elements and their likely effector genes, we examined Hi-C chromatin interaction data from mesenchymal cells and compared the HS-associated variants with regions of chromatin accessibility in human scalp cell types (Appendix S1).^38,40^

### Transcriptional reporter assays

We performed transcriptional reporter assays in the HaCaT cell line to test allelic differences in transcriptional activity. DNA fragments spanning rs981625 or a three-variant haplotype (rs17226067, rs17825774, rs17825799) were cloned into a luciferase reporter vector and transfected into HaCaT cells (Table S3; see Supporting Information). Luciferase and *Renilla* activities were measured 48 h after transfection, and the differences in activity between alleles were tested using two-tailed Student’s *t*-tests (Appendix S1).

## Results

### Identification of hidradenitis suppurativa association signals

**Figure 1:**
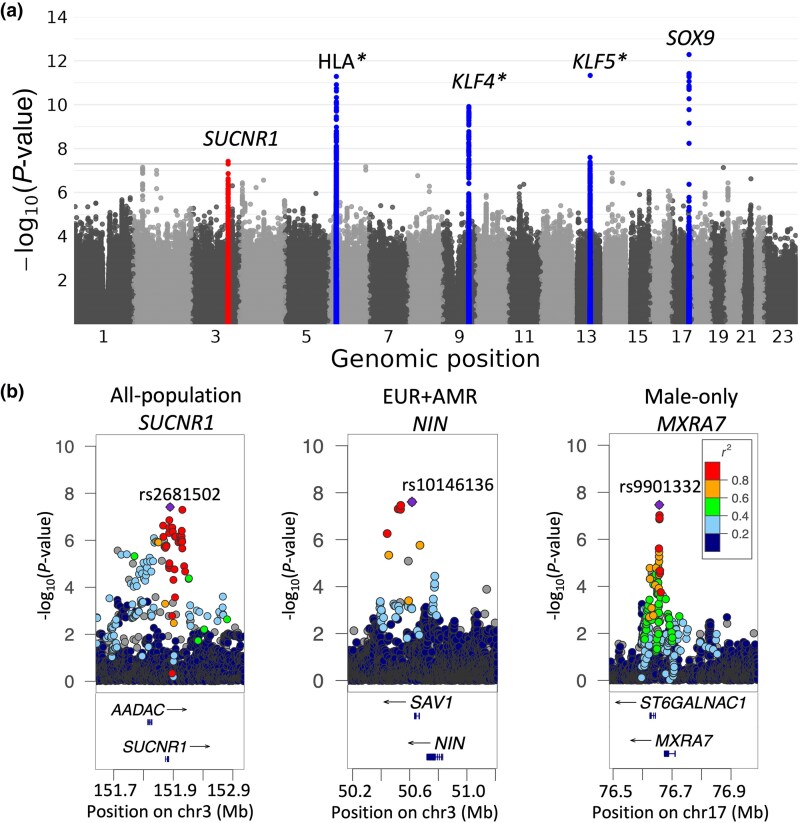
Hidradenitis suppurativa (HS) genetic association signals. (a) Manhattan plot of the associations between variants and HS in all populations. Variants within 1 Mb of a previously known HS locus are coloured in blue; variants within 1 Mb of a new HS locus (*P* ≤ 5 × 10^–8^) are coloured in red. Loci with asterisks (*) represent multi-signal loci. (b) Variant associations for new HS loci: (left) a signal near *SUCNR1* was significantly associated with HS in the all-population analysis; (middle) a signal near *NIN* was significantly associated with HS in the EUR + AMR stratified analysis; (right) a signal near *MXRA7* was significantly associated with HS in men. Chr, chromosome; EUR + AMR, European and admixed American populations.

In a GWAS meta-analysis of all participants, we identified five genome-wide significant loci (*P*-value < 5 × 10^–8^), including two we previously reported near *SOX9* and *KLF5*, two that others previously reported on chromosomes 6 and 9, respectively,^14^ and an additional locus near *SUCNR1* on chromosome 3 [Figure 1, Table 1; Table S4, Figures S1–S6 (see Supporting Information)].

**Table 1:** Eleven lead variants associated with hidradenitis suppurativa at seven loci

Chromosome	Nearby gene	Population	rsID	Alleles^a^	EAF	OR	*P*-value	Neff
Previously reported signals								
17	*SOX9*	ALL	rs17226067	G/A	0.16	1.28	5.21 × 10^–13^	17 274
13	*KLF5*	ALL	rs981625	C/G	0.95	1.55	4.68 × 10^–12^	15 521
6	HLA	ALL	rs6457592	C/T	0.53	1.19	5.18 × 10^–12^	17 274
9	*KLF4*	ALL	rs1994799	A/G	0.44	1.17	1.22 × 10^–10^	17 274
Additional loci								
3	*SUCNR1*	ALL	rs2681502	C/T	0.06	1.35	3.87 × 10^–8^	16 933
14	*NIN*	EUR + AMR	rs10146136	A/G	0.13	1.25	2.47 × 10^–8^	13 062
17	*MXRA7*	Men only	rs9901332	T/C	0.61	1.54	3.42 × 10^–8^	2266
Additional signals at established loci								
9	*KLF4*	ALL	rs1535620	G/A	0.49	1.17	7.44 × 10^–10^	16 933
6	HLA	EUR + AMR	rs9265701	A/G	0.47	1.19	9.50 × 10^–9^	8903
13	*KLF5*	EUR + AMR	rs9796066	C/T	0.37	1.18	1.42 × 10^–9^	13 062
13	*KLF5*	EUR + AMR	rs12100281	A/G	0.91	1.31	1.82 × 10^–8^	13 062

Population indicates samples included in the genome-wide analysis studies. For nonprimary signals, the *P*-values are from Genome-wide Complex Trait Analysis (GCTA) conditional analyses. Chr, chromosome; EAF, effect allele frequency; EUR + AMR, European and admixed American populations; Neff, effective sample size; OR, odds ratio [exp(β)]. ^a^Effect allele/noneffect allele.

**Figure 2:**
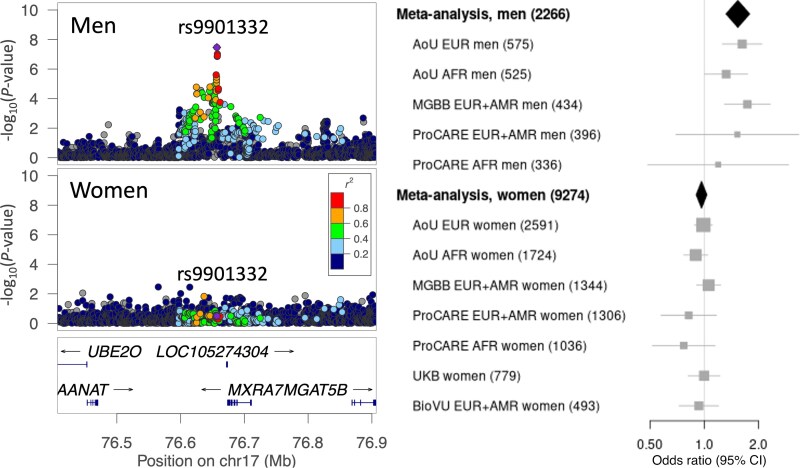
Hidradenitis suppurativa locus detected in men-only genome-wide association studies (GWAS). (Left) Variants associated with HS in men (top) vs. women (bottom). (Right) Forest plot of men-only and women-only GWAS results. The *MXRA7* locus is strong in men and shows consistent direction and strength of effect across all contributing studies. An effect is not detected in any women-only or sex-combined GWAS results. AFR, Black/African population; AoU, All of Us; EUR + AMR, European and admixed American populations; BioVU, BioVU project at Vanderbilt University Medical Center; Chr, chromosome; CI, confidence interval; MGBB, Mass General Brigham Biobank; ProCARE, Program for Research and Care Excellence; UKB, UK Biobank.

We then performed population- and sex-stratified GWAS meta-analyses. In a EUR + AMR analysis, we found a sixth locus near *NIN* [*P*-value = 2.5 × 10^–8^; Figure 1, Table 1 (Figures S7, S8; see Supporting Information)]. The lead variant, rs10146136, is rare in African populations [1000 Genomes minor allele frequency (MAF) = 0.005] but common in European populations (1000 Genomes MAF = 0.133). While we detected the variant in three AFR studies, it did not meet the significance threshold in the full meta-analysis (*P*-value = 1.3 × 10^–7^) (Figure S9; see Supporting Information). In the men-only HS analyses, we found a seventh locus near *MXRA7* (rs9901332, *P*-value = 3.4 × 10^–8^) [Figures 1, 2, Table 1; Table S4, Figure S10 (see Supporting Information)]. The lead variant’s effect size in men (β = 0.43, SE = 0.08) was comparable to the strongest effect of any other lead variant (e.g. rs981625 near *KLF5*; β = 0.44, SE = 0.06), but it had no observable effect in women, despite a fourfold larger sample size (β = –0.04, SE = 0.04, *P* = 0.32). By leveraging population- and sex-stratified analyses, we reinforced the role of common genetic variants in HS pathogenesis while identifying two additional signals that may help elucidate the genetic basis of HS.

To discover additional genetic variants that influence the risk of HS, we performed approximate conditional analyses on all seven loci.^17,18^ In the all-population meta-analysis, we identified a second signal on chromosome 9 near *KLF4* [Table 1; Table S5, Figure S11 (see Supporting Information)]. The EUR + AMR summary data confirmed this signal and revealed three more: a second signal on chromosome 6 near the HLA region (Figures S12, S13; see Supporting Information) and two additional signals near *KLF5* (Table S5, Figures S14–S17; see Supporting Information). The HLA and *KLF5* nonprimary signals narrowly missed the significance threshold in the all-population analysis (conditional *P*-values ≤ 1.7 × 10^–7^). Across all analyses, we found 11 signals in 7 loci.

### Fine-mapping

We identified credible sets of plausibly causal variants for our HS signals. We identified 9 credible sets that matched with 9 of the 11 HS association signals (Table S6; see Supporting Information); the two HLA signals did not converge. The primary *KLF5* credible set consisted of a single variant (rs981625) in low LD with the previous lead (rs17090189 LD *r^2^* = 0.24 in EUR subset of UK Biobank). While the *NIN* signal and the nonprimary *KLF5* signals narrowly missed genome-wide significance in the all-population meta-analysis (1.7 × 10^–7^ ≤ *P*-values = 1.3 × 10^–7^), they were detected in the all-population meta-analysis using fine-mapping, suggesting that the three signals were not exclusive to the population-stratified results.

As the extent of LD is typically shorter in AFR populations, we compared credible set sizes from all-population vs. EUR + AMR data. Five of eight credible sets were smaller in the all-population analysis, two were the same size and only one credible set, near *NIN*, yielded a smaller credible set with the EUR + AMR data. While differences in variant representation across studies could also influence credible set size, all EUR-specific credible set variants were well represented in the full summary statistics from the all-population analysis. These results highlight the value of multipopulation meta-analyses, even when under-represented populations do not yield population-specific signals.

### Genetic correlation with other complex traits

**Figure 3:**
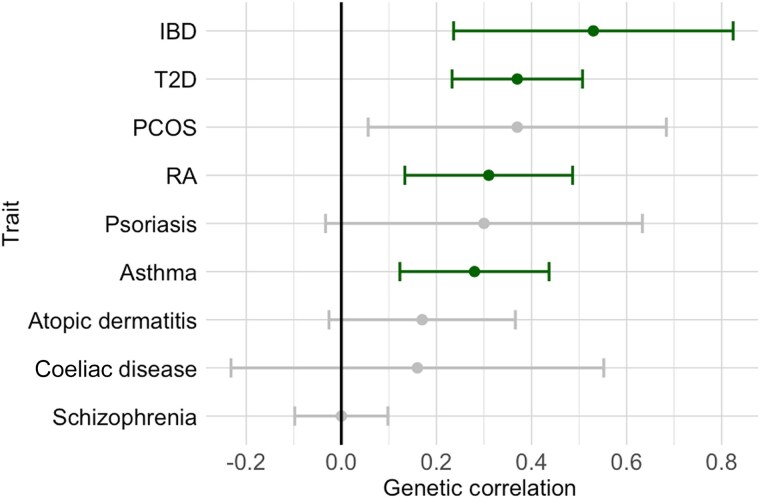
Genetic correlation between hidradenitis suppurativa and other complex traits. Green indicates Bonferroni significant correlations. IBD, inflammatory bowel disease; T2D, type 2 diabetes; PCOS, polycystic ovarian syndrome; RA, rheumatoid arthritis.

To better understand the genetic relationships between HS and other complex traits, we tested for genetic correlation. We established a significant genetic correlation (rg) between HS and IBD (rg = 0.53, *P* = 6.0 × 10^–4^), RA (rg = 0.31, *P* = 6.0 × 10^–4^), T2D (rg = 0.37, P = 1.1 × 10^–7^) and asthma (rg = 0.28, *P* = 4.0 × 10^–4^) [Figure 3; Table S7 (see Supporting Information)].^23,25,26,30^ As a negative control, schizophrenia showed no evidence of correlation (rg = –0.002, *P* = 0.97). We also found that the lead variant for the primary *KLF5* signal, rs981625, is also the lead variant for related traits including eczema, asthma, hay fever and allergic rhinitis (UK Biobank traits; http://www.nealelab.is/uk-biobank/).^30,41,42^

### Candidate genes and regulatory elements

**Figure 4:**
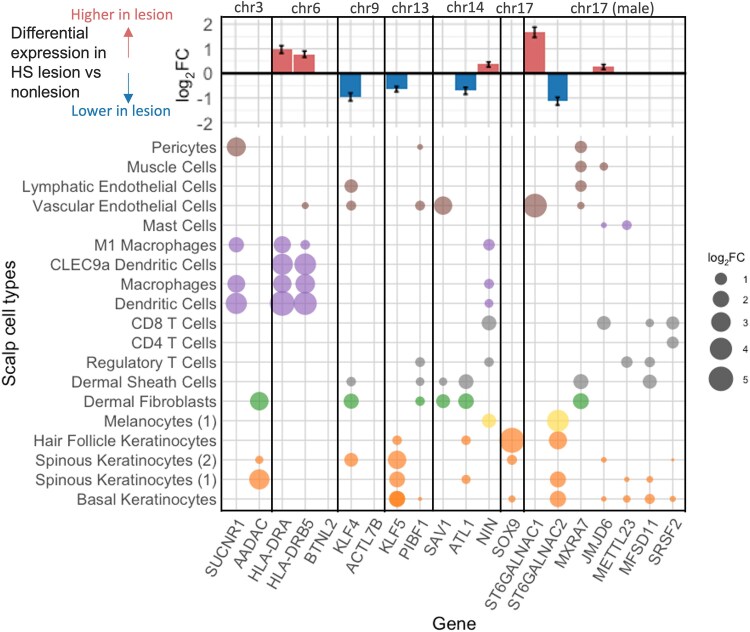
Differential expression of prioritized candidate genes in lesional vs. nonlesional tissue and in scalp cell types. We prioritized 20 genes that were within 100 kb of a lead variant, or the nearest protein-coding gene if none were within 100 kb, and genes with external evidence of relevance to hidradenitis suppurativa (HS). (Top) Log_2_ fold change (FC) of gene expression in HS lesional vs. matched nonlesional tissue. (Bottom) Log_2_ FC of gene expression in scalp cell types. Only increased expression in a cell type is shown. To be included, the cell type must be more highly expressed in at least one prioritized gene. Circles show the size of the fold change. Orange = keratinocytes; yellow = melanocytes; green = fibroblasts; purple = immune cells; brown = muscle or endothelial cells. Chr, chromosome

**Figure 5:**
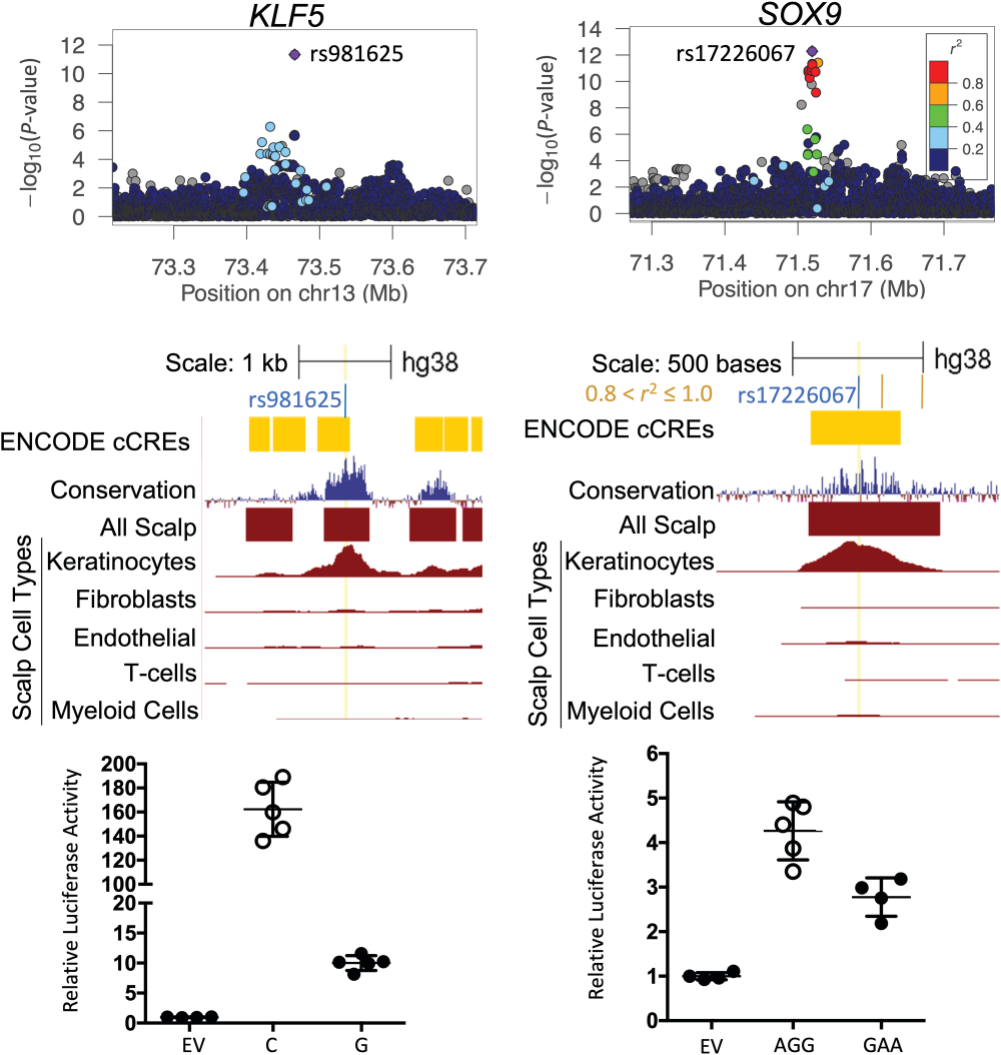
Hidradenitis suppurativa (HS) loci (left) *KLF5* and (right) *SOX9* may influence expression levels of their target genes in keratinocytes via a regulatory element that appears to be only accessible in keratinocytes. (Top row) Variant associations with HS for the two signals. (Middle left) Genome browser plots showing scalp chromatin accessibility by cell type. The lead variant of *KLF5* (rs981625) is located in a keratinocyte-accessible regulatory element that was not seen in other examined cell types (chr13_73465564_73466065) and is strongly conserved across vertebrates. No other linkage disequilibrium (LD) proxies of the lead variant are located in accessible chromatin. *KLF5* was more strongly expressed in keratinocytes than in any other cell type [log_2_fold change (FC) = 0.56–1.93 for keratinocyte cell clusters, *P*-values ≤ 4 × 10^–166^]. (Bottom left) The rs981625-C allele, which was associated with higher HS risk, showed 10-fold greater transcriptional activity than the rs981625-G reference allele (*P* = 2.9 × 10^–7^). (Middle right) The *SOX9* lead variant rs17226067, and its LD proxies rs17825774 and rs17825799 (*r^2^* = 1, shown in gold) are located in a region of open chromatin detected in keratinocytes but not in other surveyed cell types (chr17_71519287_71519788); no other LD proxies are located in accessible chromatin. *SOX9* was also more highly expressed in hair follicle keratinocyte clusters than other scalp cell types (log_2_FC = 0.28–5.19, *P*-values ≤ 5 × 10^–86^). (Bottom right) The three-variant risk haplotype of rs17226067-G, rs17825774-A and rs17825799-A alleles showed more than twofold reduced transcriptional activity vs. the reference AGG haplotype (*P* = 0.001). cCRE, candidate cis-regulatory element; Chr, chromosome; EV, empty vector.

To prioritize candidate genes and to identify potential regulatory elements affected by HS risk variants, we integrated GWAS findings with variant annotations and transcriptomic, eQTL, chromatin accessibility and Hi-C data (Tables S8–S14; see Supporting Information). We strengthened the evidence for *KLF5* and *SOX9* as candidate genes. *KLF5* was less expressed in HS lesions vs. unaffected skin [log_2_ fold change (FC) = –0.6, *P* = 2.9 × 10^–9^] and exhibited high gene activity in scalp keratinocyte clusters (log_2_FC 0.6–2.6, *P* ≤ 4.0 × 10^–166^) but no other cell type (Figure 4; Table S10). The *KLF5* primary signal lead variant was in a regulatory element that appears to be only accessible in keratinocytes (Figure 5; Table S13). *SOX9* was highly expressed in hair follicle, spinous and basal keratinocyte cell clusters (log_2_FC 0.3–5.2, *P* ≤ 5 × 10^–86^) but no other scalp cell type (Figure 4; Table S10). The *SOX9* lead and LD proxies were also in a regulatory region showing chromatin accessibility in keratinocytes but not in other profiled cell types (Figure 5; Table S13). These findings add support to the hypothesis that *KLF5* and *SOX9* signals could act through a keratinocyte-accessible regulatory element not observed in other cell types examined to influence *KLF5* and *SOX9* expression levels in keratinocytes.

The two chromosome 6 signals were near *HLA-DRA* and *HLA-DRB5*. Both genes were highly expressed in immune-relevant cell type clusters, including dendritic cells and macrophages, and were more highly expressed in HS lesional than nonlesional tissue (Figure 4; Table S10). In addition, all proxies for the second HLA signal were in one regulatory element that appears to be only accessible in lymphoids (Table S13), consistent with the known role of HLA genes in immunity.

**Figure 6:**
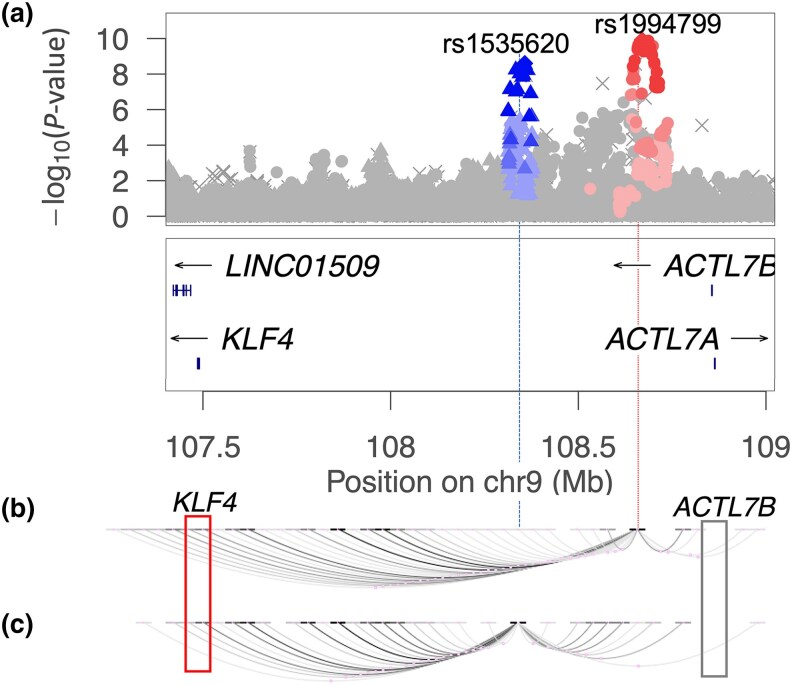
Both conditionally distinct signals on chromosome 9 exhibit a significant Hi-C chromatin interaction peak in mesenchymal data. (a) Variants associated with hidradenitis suppurativa (HS) at two conditionally distinct signals. (b) Mesenchymal cell Hi-C data anchored around rs1994799,^40^ the lead variant for the primary HS signal. (c) Mesenchymal cell Hi-C data anchored around rs1535620, the lead variant for the secondary HS signal. The target regions immediately flanking the anchor regions and target regions with FitHiC *Q*-values ≥ 0.01 are not shown. Arc colours are scaled by strength of association measured by –log_10_(*P*-value), with the darkest lines showing the link with the strongest *P*-value. Hi-C data in the red box, centred around *KLF4*, showed significant interactions; no significant interactions were observed around the more proximal gene *ACTL7B* (grey box). Chr, chromosome.

Mesenchymal Hi-C chromatin interaction data have suggested long-range regulatory effects for lead variants on chromosomes 14 and 9.^40^ rs10146136 on chromosome 14 interacted with *NIN* (Figure S18; see Supporting Information), suggesting a regulatory link to *NIN* [107 kb from the transcription start site (TSS)]. *NIN* showed increased expression in HS lesions (Figure 4; Table S10). Similarly, both lead variants on chromosome 9 (rs1994799 and rs1535620) interacted with the promoter of *KLF4* [Figure 6; Figure S19 (see Supporting Information)], despite 900 kb between the leads and the *KLF4* TSS. *KLF4* demonstrated decreased expression in HS lesions [log_2_FC = –0.96, *P* = 1.0 × 10^–10^ (Figure 4; Table S10)]. Hi-C links were not observed in mesenchymal for the closer *ACTL7B* (185 kb from TSS), and *ACTL7B* showed no change in lesional vs. nonlesional tissue (log_2_FC = 0.93, *P* = 0.71) (Figure 4). These findings suggested potential regulatory links between the identified variants and target genes, although further investigation is needed.

For 9 of the 11 signals, at least 1 lead or high LD proxy variant in the signal’s credible set was located within a scalp regulatory element (Table S13). These data suggest that causal variants reside within these elements, refining the set of most plausible causal variants (Table S6).

### Variant function

To investigate the allelic effects of two signals with clear lead variants, we examined regulatory function in transcriptional reporter assays in epidermal HaCaT cells. The *KLF5* rs981625-C HS risk allele showed 10-fold greater transcriptional activity than the reference allele (*P* < 2.9 × 10^–7^) [Figure 5; Figure S20 (see Supporting Information)]. At *SOX9*, a three-variant haplotype that included the lead HS risk variant rs17226067-G, as well as rs17825774-A and rs17825799-A, showed more than twofold lower transcriptional activity than the reference haplotype (*P* = 0.001) (Figure 5; Figure S20). These data suggest that rs981625 and at least one variant in the haplotype are plausible functional regulatory variants responsible for HS risk at the *SOX9* and *KLF5* signals.

## Discussion

We performed a meta-analysis of 4540 cases of HS, identifying 11 HS signals in 7 loci. We identified five loci in the all-population meta-analysis, an additional locus in a EUR + AMR analysis and a locus in the men-only analysis. Four loci overlapped known regions (*SOX9*, *KLF5*, *KLF4* and HLA), while three were novel: *SUCNR1*, *NIN* (EUR + AMR-only locus) and *MXRA7* (male-only locus). Collectively, these results highlight the complexity of HS genetics, the importance of considering population structure and the potential of sex differences in HS aetiology.

We established a genetic correlation between HS and four common inflammatory and metabolic conditions: IBD, RA, T2D and asthma.^23,25,26,30^ The correlation with IBD was the strongest; patients with IBD were nine times more likely to develop HS than the general population,^43^ suggesting that overlapping immune and inflammatory mechanisms may contribute to both conditions. Rare variants in *NOD2*, a gene that has been widely implicated in Crohn disease,^44^ have been associated with HS.^45^ While many of these variants have been classified as benign or likely benign *in silico*,^46^ one variant (rs104895452) has a high probability of being deleterious *in silico*.^45,47^ HS and IBD are often successfully treated with tumour necrosis factor (TNF)-α inhibitors,^48,49^ highlighting how shared genetic architecture can inform therapeutic strategies. Further, rs981625, the lead variant at *KLF5*, was also the lead variant for eczema, asthma, hay fever and allergic rhinitis.^30,41,42^ Understanding these shared genetic bases could offer insights into common pathophysiological mechanisms and potentially guide future research toward more integrated therapeutic strategies.

Previously, we described how dysregulation of *KLF5* and *SOX9* could be linked to the perturbations in follicular and epidermal differentiation, inflammation and wound healing that are characteristic of HS. Here, we identified likely causal variants driving these noncoding signals. The *KLF5* rs981625-C allele was associated with greater transcriptional activity and higher HS risk, aligning with mouse studies where *Klf5* overexpression led to hyperkeratosis and follicular occlusions.^50^ Similarly, a *SOX9* haplotype including the rs17226067-G allele was associated with reduced transcriptional activity and increased risk of HS, consistent with the observation that mice deficient in *Sox9* exhibit follicular degeneration, epidermal hyperplasia and dermal scarring.^51^

Chromatin interaction data implicated *KLF4* as a target gene for two chromosome 9 signals.^40^ A transcription factor linked to psoriasis,^52^ Krüppel-like factor 4 (KLF4) regulates wound healing, skin barrier function, proliferation and follicular/epidermal differentiation.^53^ It is both regulated by and regulates *NOTCH1* signalling and is induced by inflammatory cytokines.^54,55^ KLF4 stimulates production of interleukin (IL)-36 and IL-8, both potent inducers of neutrophil-mediated inflammation, as well as IL-6 and IL-10. In human keratinocyte knockdown models of *NCSTN*, a γ-secretase family member that is mutated in some cases of familial HS, *KLF4* was upregulated, particularly in the presence of *Staphylococcus aureus*, consistent with the role of bacteria in potentiating inflammation in HS.^56^ Overexpression of *Klf4* in mice led to impaired differentiation of follicular epithelium with impaired hair growth.^57^ Given its similarities to SRY-box transcription factor 9 (SOX9) and Krüppel-like factor 5 (KLF5) in follicular/epidermal differentiation, wound healing and inflammation, KLF4 may also influence HS pathogenesis.

We also identified plausible candidate genes at our other loci. *HLA-DRA*, which is upregulated in HS lesions, regulates antigen presentation and may contribute to inflammation via dysregulated host–microbiome interactions. Ninein (NIN), a microtubule regulator, is crucial for immune function, barrier function and neutrophil extracellular trap regulation.^58,59^ In sepsis, IL-6 downregulation reduced NIN in monocytes, which allowed for the persistence of pathogens and potentiation of other inflammatory bacterial responses.^60^ A similar process could contribute to dysbiosis, biofilm formation and chronic inflammation in HS tissue.^61^ Succinate receptor 1 (SUCNR1) regulates inflammasome priming and glucose homeostasis. Deficiency in *Sucnr1* is linked not only to obesity, glucose intolerance and enhanced inflammatory signalling,^62^ but also to reduced inflammasome activation in mouse colitis models, suggesting it may also play a proinflammatory role in some settings.^63^ In macrophages, *KLF4* knockdown has been associated with reduced expression of *SUCNR1*,^62^ potentially linking these risk loci together in HS pathogenesis. Finally, matrix remodelling associated 7 (MXRA7) may protect against propsoriasis cytokines,^64^ as mouse models deficient in *Mxra7* develop more severe disease. Loss of *MXRA7* resulted in epidermal proliferation increases, expression of TNF-α, IL-6 and IL-1, and impaired wound healing,^65^ all of which are linked to HS pathogenesis. However, many genes near *MXRA7* could contribute to risk. Further studies are needed to investigate these and other nearby genes for their roles in HS pathogenesis.

This GWAS meta-analysis has limitations. Firstly, due to sample size constraints, we examined, at most, two population groups per cohort and analysed people together from European and admixed American populations (EUR + AMR). The results cannot be interpreted to infer racial or ethnic differences. Most signals were not significant in the AFR GWAS (0.005 ≤ *P*-value ≤ 0.78), highlighting the need for larger AFR cohorts with HS to identify additional HS GWAS signals and improve fine-mapping. Allele frequency differences affect association detection; all but one lead variant (*SOX9* lead variant rs17226067) was less common in AFR vs. EUR + AMR populations. New signals, especially those with different effects across populations, should be validated. Secondly, as individuals with HS experience an average of 7–10 years between the manifestation of the first symptoms until a formal diagnosis is made,^66,67^ some control participants may have HS, biasing the results towards the null. Thirdly, we found only one co-localization between HS signals and eQTLs; as eQTL studies expand, we expect to uncover further HS-eQTL co-localizations that could enhance understanding of HS pathogenesis. Fourthly, this study focused on common variants (MAF ≥ 1%). HS is known to be associated with rare variants;^45^ integrating rare variant analyses with future common genetic association studies would enhance our understanding of its genetic basis.

Overall, we identified potential mechanisms and candidate genes for HS by stratifying analyses by population and sex, conducting conditional analyses and integrating HS signals with complementary GWAS traits, expression and regulatory data, and studies of variant function. This work represents one of the largest HS meta-analyses to date (effective sample size, *n* = 17 244) and the largest meta-analysis in AFR participants (effective sample size, *n* = 4212). While further research with larger and more diverse cohorts is needed to fully elucidate the genetic contributors to HS, our findings provide valuable insights into disease pathology and may inform the development of novel therapeutics.

## Supplementary Material

ljaf277_Supplementary_Data

## Data Availability

Upon publication, the data underlying this article will be available in the GWAS Catalog, at https://www.ebi.ac.uk/gwas/. Genotype-Tissue Expression (GTEx) summary results can be found at https://console.cloud.google.com/storage/browser/gtex-resources.

## References

[1] van Straalen KR, Prens EP, Willemsen G et al Contribution of genetics to the susceptibility to hidradenitis suppurativa in a large, cross-sectional Dutch twin cohort. JAMA Dermatol 2020; 156:1359–62. 33052394 10.1001/jamadermatol.2020.3630 PMC7557497

[2] Kjaersgaard Andersen R, Clemmensen SB, Larsen LA et al Evidence of gene-gene interaction in hidradenitis suppurativa: a nationwide registry study of Danish twins. Br J Dermatol 2022; 186:78–85. 34289077 10.1111/bjd.20654

[3] Bruinsma RL, Fajgenbaum K, Yang Y et al Assessment of familial risk in patients with hidradenitis suppurativa. Br J Dermatol 2021; 184:753–4. 33152101 10.1111/bjd.19664 PMC8035158

[4] Vinkel C, Thomsen SF. Hidradenitis suppurativa: causes, features, and current treatments. J Clin Aesthet Dermatol 2018; 11:17–23. 30519375 PMC6239161

[5] Garg A, Malviya N, Strunk A et al Comorbidity screening in hidradenitis suppurativa: Evidence-based recommendations from the US and Canadian Hidradenitis Suppurativa Foundations. J Am Acad Dermatol 2022; 86:1092–101. 33493574 10.1016/j.jaad.2021.01.059 PMC8298595

[6] Phan K, Tatian A, Woods J et al Prevalence of inflammatory bowel disease (IBD) in hidradenitis suppurativa (HS): systematic review and adjusted meta-analysis. Int J Dermatol 2020; 59:221–8. 31631340 10.1111/ijd.14697

[7] Phan K, Charlton O, Smith SD. Hidradenitis suppurativa and polycystic ovarian syndrome: systematic review and meta-analysis. Australas J Dermatol 2020; 61:e28–33. 31261440 10.1111/ajd.13110

[8] van Straalen KR, Prens EP, Gudjonsson JE. Insights into hidradenitis suppurativa. J Allergy Clin Immunol 2022; 149:1150–61. 35189127 10.1016/j.jaci.2022.02.003

[9] Maronese CA, Moltrasio C, Marzano AV. Hidradenitis suppurativa-related autoinflammatory syndromes: an updated review on the clinics, genetics, and treatment of pyoderma gangrenosum, acne and suppurative hidradenitis (PASH), pyogenic arthritis, pyoderma gangrenosum, acne and suppurative hidradenitis (PAPASH), synovitis, acne, pustulosis, hyperostosis and osteitis (SAPHO), and rarer forms. Dermatol Clin 2024; 42:247–65. 38423685 10.1016/j.det.2023.12.004

[10] Pink AE, Simpson MA, Desai N et al γ-Secretase mutations in hidradenitis suppurativa: new insights into disease pathogenesis. J Invest Dermatol 2013; 133:601–7. 23096707 10.1038/jid.2012.372

[11] Balić A, Marinović B, Bukvić Mokos Z. The genetic aspects of hidradenitis suppurativa. Clin Dermatol 2023; 41:551–63. 37652193 10.1016/j.clindermatol.2023.08.022

[12] Morales-Heil DJ, Cao L, Sweeney C et al Rare missense variants in the SH3 domain of PSTPIP1 are associated with hidradenitis suppurativa. HGG Adv 2023; 4:100187. 37013170 10.1016/j.xhgg.2023.100187 PMC10066561

[13] Metpally RP, Vishweswaraiah S, Krishnamurthy S et al Identification of novel genetic risk variants associated with hidradenitis suppurativa in an exome sequencing cohort of 92,455 individuals. Dermatology 2024; 240:739–49. 39396498 10.1159/000540359

[14] Kjærsgaard Andersen R, Stefansdottir L, Riis PT et al A genome-wide association meta-analysis links hidradenitis suppurativa to common and rare sequence variants causing disruption of the Notch and Wnt/β-catenin signaling pathways. J Am Acad Dermatol 2025; 92:761–72. 39645042 10.1016/j.jaad.2024.11.050

[15] Sun Q, Broadaway KA, Edmiston SN et al Genetic variants associated with hidradenitis suppurativa. JAMA Dermatol 2023; 159:930–8. 37494057 10.1001/jamadermatol.2023.2217 PMC10372759

[16] Willer CJ, Li Y, Abecasis GR. METAL: fast and efficient meta-analysis of genomewide association scans. Bioinformatics 2010; 26:2190–1. 20616382 10.1093/bioinformatics/btq340 PMC2922887

[17] Yang J, Lee SH, Goddard ME, Visscher PM. GCTA: a tool for genome-wide complex trait analysis. Am J Hum Genet 2011; 88:76–82. 21167468 10.1016/j.ajhg.2010.11.011 PMC3014363

[18] Yang J, Ferreira T, Morris AP et al Conditional and joint multiple-SNP analysis of GWAS summary statistics identifies additional variants influencing complex traits. Nat Genet 2012; 44:369–75. 22426310 10.1038/ng.2213 PMC3593158

[19] Zou Y, Carbonetto P, Wang G, Stephens M. Fine-mapping from summary data with the “Sum of Single Effects” model. PLOS Genet 2022; 18:e1010299. 35853082 10.1371/journal.pgen.1010299 PMC9337707

[20] Bulik-Sullivan B, Finucane HK, Anttila V et al An atlas of genetic correlations across human diseases and traits. Nat Genet 2015; 47:1236–41. 26414676 10.1038/ng.3406 PMC4797329

[21] Bulik-Sullivan BK, Loh P-R, Finucane HK et al LD score regression distinguishes confounding from polygenicity in genome-wide association studies. Nat Genet 2015; 47:291–5. 25642630 10.1038/ng.3211 PMC4495769

[22] Jiang L, Zheng Z, Fang H, Yang J A generalized linear mixed model association tool for biobank-scale data. Nat Genet 2021; 53:1616–21. 34737426 10.1038/s41588-021-00954-4

[23] Liu JZ, van Sommeren S, Huang H et al Association analyses identify 38 susceptibility loci for inflammatory bowel disease and highlight shared genetic risk across populations. Nat Genet 2015; 47:979–86. 26192919 10.1038/ng.3359 PMC4881818

[24] Day F, Karaderi T, Jones MR et al Large-scale genome-wide meta-analysis of polycystic ovary syndrome suggests shared genetic architecture for different diagnosis criteria. PLOS Genet 2018; 14:e1007813. 30566500 10.1371/journal.pgen.1007813 PMC6300389

[25] Saevarsdottir S, Stefansdottir L, Sulem P et al Multiomics analysis of rheumatoid arthritis yields sequence variants that have large effects on risk of the seropositive subset. Ann Rheum Dis 2022; 81:1085–95. 35470158 10.1136/annrheumdis-2021-221754 PMC9279832

[26] Suzuki K, Hatzikotoulas K, Southam L et al Genetic drivers of heterogeneity in type 2 diabetes pathophysiology. Nature 2024; 627:347–57. 38374256 10.1038/s41586-024-07019-6 PMC10937372

[27] Budu-Aggrey A, Kilanowski A, Sobczyk MK et al European and multi-ancestry genome-wide association meta-analysis of atopic dermatitis highlights importance of systemic immune regulation. Nat Commun 2023; 14:6172. 37794016 10.1038/s41467-023-41180-2 PMC10550990

[28] Sakaue S, Kanai M, Tanigawa Y et al A cross-population atlas of genetic associations for 220 human phenotypes. Nat Genet 2021; 53:1415–24. 34594039 10.1038/s41588-021-00931-x PMC12208603

[29] Trubetskoy V, Pardiñas AF, Qi T et al Mapping genomic loci implicates genes and synaptic biology in schizophrenia. Nature 2022; 604:502–8. 35396580 10.1038/s41586-022-04434-5 PMC9392466

[30] Tsuo K, Zhou W, Wang Y et al Multi-ancestry meta-analysis of asthma identifies novel associations and highlights the value of increased power and diversity. Cell Genom 2022; 2:100212. 36778051 10.1016/j.xgen.2022.100212 PMC9903683

[31] Cerezo M, Sollis E, Ji Y et al The NHGRI-EBI GWAS Catalog: standards for reusability, sustainability and diversity. Nucleic Acids Res 2025; 53:D998–1005. 39530240 10.1093/nar/gkae1070 PMC11701593

[32] Liu X, White S, Peng B et al WGSA: an annotation pipeline for human genome sequencing studies. J Med Genet 2016; 53:111–12. 26395054 10.1136/jmedgenet-2015-103423 PMC5124490

[33] GTEx Consortium . The GTEx Consortium atlas of genetic regulatory effects across human tissues. Science 2020; 369:1318–30. 32913098 10.1126/science.aaz1776 PMC7737656

[34] Shore CJ, Villicaña S, El-Sayed Moustafa J et al Genetic effects on the skin methylome in healthy older twins. Am J Hum Genet 2024; 111:1932–52. 39137780 10.1016/j.ajhg.2024.07.010 PMC11393713

[35] Kerimov N, Hayhurst JD, Peikova K et al A compendium of uniformly processed human gene expression and splicing quantitative trait loci. Nat Genet 2021; 53:1290–9. 34493866 10.1038/s41588-021-00924-w PMC8423625

[36] Võsa U, Claringbould A, Westra H-J et al Large-scale cis- and trans-eQTL analyses identify thousands of genetic loci and polygenic scores that regulate blood gene expression. Nat Genet 2021; 53:1300–10. 34475573 10.1038/s41588-021-00913-z PMC8432599

[37] Xiao Q, Mears J, Nathan A et al Immunosuppression causes dynamic changes in expression QTLs in psoriatic skin. Nat Commun 2023; 14:6268. 37805522 10.1038/s41467-023-41984-2 PMC10560299

[38] Ober-Reynolds B, Wang C, Ko JM et al Integrated single-cell chromatin and transcriptomic analyses of human scalp identify gene-regulatory programs and critical cell types for hair and skin diseases. Nat Genet 2023; 55:1288–300. 37500727 10.1038/s41588-023-01445-4 PMC11190942

[39] Freudenberg JM, Liu Z, Singh J et al A Hidradenitis suppurativa molecular disease signature derived from patient samples by high-throughput RNA sequencing and re-analysis of previously reported transcriptomic data sets. PLOS ONE 2023; 18:e0284047. 37023004 10.1371/journal.pone.0284047 PMC10079067

[40] Schmitt AD, Hu M, Jung I et al A compendium of chromatin contact maps reveals spatially active regions in the human genome. Cell Rep 2016; 17:2042–59. 27851967 10.1016/j.celrep.2016.10.061 PMC5478386

[41] Johansson Å, Rask-Andersen M, Karlsson T, Ek WE. Genome-wide association analysis of 350 000 Caucasians from the UK Biobank identifies novel loci for asthma, hay fever and eczema. Hum Mol Genet 2019; 28:4022–41. 31361310 10.1093/hmg/ddz175 PMC6969355

[42] Kichaev G, Bhatia G, Loh P-R et al Leveraging polygenic functional enrichment to improve GWAS power. Am J Hum Genet 2019; 104:65–75. 30595370 10.1016/j.ajhg.2018.11.008 PMC6323418

[43] Yadav S, Singh S, Varayil JE et al Hidradenitis suppurativa in patients with inflammatory bowel disease: a population-based cohort study in Olmsted County, Minnesota. Clin Gastroenterol Hepatol 2016; 14:65–70. 25952308 10.1016/j.cgh.2015.04.173 PMC4635068

[44] Ashton JJ, Seaby EG, Beattie RM, Ennis S. NOD2 in Crohn’s disease – unfinished business. J Crohns Colitis 2023; 17:450–8. 36006803 10.1093/ecco-jcc/jjac124 PMC10069614

[45] Muret K, Le Goff V, Dandine-Roulland C et al Comprehensive catalog of variants potentially associated with hidradenitis suppurativa, including newly identified variants from a cohort of 100 patients. Int J Mol Sci 2024; 25:10374. 39408704 10.3390/ijms251910374 PMC11476843

[46] Mintoff D, Borg I, Pace NP. NOD2 at the interface of hidradenitis suppurativa and inflammatory bowel disease – an *in silico* analysis. Exp Dermatol 2024; 33:e14928. 37694954 10.1111/exd.14928

[47] de Oliveira ASLE, Bloise G, Moltrasio C et al Transcriptome meta-analysis confirms the hidradenitis suppurativa pathogenic triad: upregulated inflammation, altered epithelial organization, and dysregulated metabolic signaling. Biomolecules 2022; 12:1371. 36291580 10.3390/biom12101371 PMC9599370

[48] Savage KT, Flood KS, Porter ML, Kimball AB. TNF-α inhibitors in the treatment of hidradenitis suppurativa. Ther Adv Chronic Dis 2019; 10:2040622319851640. 10.1177/2040622319851640 PMC6540495 31191873

[49] Souza RF, Caetano MAF, Magalhães HIR, Castelucci P. Study of tumor necrosis factor receptor in the inflammatory bowel disease. World J Gastroenterol 2023; 29:2733–46. 37274062 10.3748/wjg.v29.i18.2733 PMC10237104

[50] Sur I, Rozell B, Jaks V et al Epidermal and craniofacial defects in mice overexpressing Klf5 in the basal layer of the epidermis. J Cell Sci 2006; 119:3593–601. 16912082 10.1242/jcs.03070

[51] Vidal VPI, Chaboissier M-C, Lützkendorf S et al Sox9 is essential for outer root sheath differentiation and the formation of the hair stem cell compartment. Curr Biol 2005; 15:1340–51. 16085486 10.1016/j.cub.2005.06.064

[52] Ray-Jones H, Duffus K, McGovern A et al Mapping DNA interaction landscapes in psoriasis susceptibility loci highlights *KLF4* as a target gene in 9q31. BMC Biol 2020; 18:47. 32366252 10.1186/s12915-020-00779-3 PMC7199343

[53] Ghaleb AM, Yang VW. Krüppel-like factor 4 (KLF4): What we currently know. Gene 2017; 611:27–37. 28237823 10.1016/j.gene.2017.02.025 PMC5391259

[54] Lebson L, Gocke A, Rosenzweig J et al Cutting edge: the transcription factor Kruppel-like factor 4 regulates the differentiation of Th17 cells independently of RORγt. J Immunol 2010; 185:7161–4. 21076063 10.4049/jimmunol.1002750 PMC3071015

[55] Yin J, Xie X, Yao J et al Transcription factor Krüppel-like factor 4 upregulated G protein-coupled receptor 30 alleviates intestinal inflammation and apoptosis, and protects intestinal integrity from intestinal ischemia-reperfusion injury. Immun Inflamm Dis 2023; 11:e940. 37506161 10.1002/iid3.940 PMC10373568

[56] Zhang Y, Jia W, Wang X et al Inflammatory loop involving *Staphylococcus aureus*, IL-36γ, and cathepsin S drives immunity disorders in familial acne inversa keratinocytes. Heliyon 2024; 10:e31509. 38947455 10.1016/j.heliyon.2024.e31509 PMC11214400

[57] Han H, Qin H, Yang Y et al Effect of overexpression of KLF4 on the growth and development of hair follicles in mice. Dev Genes Evol 2023; 233:137–45. 37561178 10.1007/s00427-023-00708-8

[58] Lecland N, Hsu C-Y, Chemin C et al Epidermal development requires ninein for spindle orientation and cortical microtubule organization. Life Sci Alliance 2019; 2:e201900373. 30923192 10.26508/lsa.201900373 PMC6441496

[59] Haider P, Kral-Pointner JB, Mayer J et al Neutrophil extracellular trap degradation by differently polarized macrophage subsets. Arterioscler Thromb Vasc Biol 2020; 40:2265–78. 32673525 10.1161/ATVBAHA.120.314883 PMC7447175

[60] Akoumianaki T, Vaporidi K, Diamantaki E et al Uncoupling of IL-6 signaling and LC3-associated phagocytosis drives immunoparalysis during sepsis. Cell Host Microbe 2021; 29:1277–93. 34214493 10.1016/j.chom.2021.06.002

[61] Chopra D, Arens RA, Amornpairoj W et al Innate immunity and microbial dysbiosis in hidradenitis suppurativa – vicious cycle of chronic inflammation. Front Immunol 2022; 13:960488. 35967376 10.3389/fimmu.2022.960488 PMC9368759

[62] Keiran N, Ceperuelo-Mallafré V, Calvo E et al SUCNR1 controls an anti-inflammatory program in macrophages to regulate the metabolic response to obesity. Nat Immunol 2019; 20:581–92. 30962591 10.1038/s41590-019-0372-7

[63] Bauset C, Lis-Lopez L, Coll S et al SUCNR1 mediates the priming step of the inflammasome in intestinal epithelial cells: relevance in ulcerative colitis. Biomedicines 2022; 10:532. 35327334 10.3390/biomedicines10030532 PMC8945150

[64] Ning J, Shen Y, Wang T et al Altered expression of matrix remodelling associated 7 (MXRA7) in psoriatic epidermis: evidence for a protective role in the psoriasis imiquimod mouse model. Exp Dermatol 2018; 27:1038–42. 29781547 10.1111/exd.13687

[65] Sun Z, Ke P, Shen Y et al MXRA7 is involved in monocyte-to-macrophage differentiation. Mol Immunol 2024; 171:12–21. 38735126 10.1016/j.molimm.2024.05.001

[66] Saunte DM, Boer J, Stratigos A et al Diagnostic delay in hidradenitis suppurativa is a global problem. Br J Dermatol 2015; 173:1546–9. 26198191 10.1111/bjd.14038

[67] Kokolakis G, Wolk K, Schneider-Burrus S et al Delayed diagnosis of hidradenitis suppurativa and its effect on patients and healthcare system. Dermatology 2020; 236:421–30. 32610312 10.1159/000508787 PMC7592906

