# Supplementary material for: Genomic loci and molecular genetic mechanisms for hidradenitis suppurativa
Source: Br J Dermatol. 2025 Jul 12;193(5):948–58. doi: 10.1093/bjd/ljaf277 (PMC12279254; doi:10.1093/bjd/ljaf277)
Supplement: ljaf277_Supplementary_Data [file ljaf277_supplementary_data.zip › Appendix S1.pdf]

Supplementary Methods

Genetic Association Analyses

We enrolled and genotyped 266 additional HS patients (total 986) into the HS Program for Research and Care Excellence (ProCARE) study at the University of North Carolina (UNC) Department of Dermatology. HS ProCARE patients were evaluated and diagnosed at outpatient dermatology clinics. Methods for genotyping, imputation, and selection of controls (**Table S1**) are described in detail elsewhere[1]; briefly, we combined new and original HS patients participating in ProCARE with controls from the National Longitudinal Study of Adolescent to Adult Health (Add Health)[2,3]. We performed genotype imputation on variants observed in both cases and controls with TOPMed freeze 8 as a reference[4–6].

Five additional studies contributed to the GWAS meta-analysis; details on each study, including genotyping array, imputation, and case definition are in **Table S1**. For the five contributing studies, HS diagnosis was defined by International Classification of Diseases, Ninth Revision (ICD-9) (705.83, Hidradenitis) or International Statistical Classification of Diseases and Related Health Problems, Tenth Revision (ICD-10) (L73.2, Hidradenitis suppurativa). Cohorts either reported variants in Genome Reference Consortium human build 38/hg38, or results were lifted from hg19 to hg38 using the UCSC LiftOver tool[7].

Each cohort used imputed genotype data and covariates to perform association analyses with HS status (**Table S1**). To control for population structure that persisted after adjusting for principal components, study analysts assigned cases and controls to two population groups: black/African/African American participants (AFR) and all other participants (**Table S1**). In the second population, over 95.5% of participants were from European populations (EUR), while 3.6% were from Admixed American (AMR) populations. While <1% represent other populations or more than one population background, for brevity we refer to the second group as EUR+AMR. GWAS were performed in each cohort separately by population group. When available, each cohort adjusted for age, sex, body mass index (BMI, calculated as weight in kilograms divided by height in meters squared), smoking status, and relatedness and population structure as appropriate for their study (**Table S1**). Each population group for each cohort excluded variants with low minor allele frequency (MAF < 0.01), low imputation quality (r^2 <0.3), or exceptionally large effect standard errors (standard error >10).

GWAS Meta-analyses

We conducted meta-analyses of GWAS results for HS from the six contributing cohorts (Table S1). We applied genomic control on summary statistics for each study prior to meta-analysis of effect size estimates and standard errors using METAL[8]. Only variants represented by two or more studies were included in the meta-analysis. We defined a locus as a lead variant with P-value < $5x10^{-8}$ and all variants within 1 Mb of the lead

variant. We extended the definition of a locus in the HLA region to variants within 2 Mb to account for extensive linkage disequilibrium (LD) in the region.

We performed a first GWAS meta-analysis by combining all population groups from all cohorts. Next, we performed analyses stratified by population group, separating AFR participants from all other participants. Lastly, we performed analyses stratified by sex. For the five cohorts for which individual level data was available (**Tables S1-S2**), we performed stratified GWAS analyses for each combination of population (AFR and all others) and sex (male and female). All listed covariates (**Table S1**) except sex were included in the sex-specific models. We excluded results from stratified analyses if the effective sample size of the stratified group was less than 300. Due to small numbers of cases in some cohorts, particularly in the male-only analysis (**Table S2**), we restricted sex-specific analyses to variants with MAF ≥ .05.

<u>Conditionally Distinct Signals</u>

A locus refers to a lead variant and all variants within 1 Mb; however, this region can harbor several genetic signals distinctly associated with HS. To identify conditionally distinct signals within a locus, we performed approximate conditional analysis using GCTA[9,10] with a collinearity threshold of 0.5 and a subset of 40,000 unrelated European-ancestry individuals from the United Kingdom Biobank (40K EUR UKB) as the LD reference. Since UKB data are mapped to hg19, we lifted our HS summary statistics back to hg19 using the UCSC LiftOver tool[7,11] prior to performing conditional analysis. For loci in which more than one signal was identified, we also used GCTA to obtain isolated summary association results, conditioning on all other lead variants in the locus. Lastly, we lifted the isolated GCTA summary results back to hg38. For all downstream analyses, unless stated otherwise, we use marginal summary statistics for single-signal loci and GCTA-isolated summary statistics for multi-signal loci.

<u>Fine-Mapping</u>

To identify likely causal variants at each locus, we used the Bayesian fine-mapping method SuSiE[12] (susieR package 0.12.35) to identify 95% credible sets. We calculated LD reference matrices of variants spanning 500 kb before the first lead variant to 500 kb after the last lead variant using PLINK[13]. For summary statistics, we input marginal all-population and marginal EUR+AMR GWAS meta-analysis data lifted back to hg19. We set the minimum absolute correlation between variants in a credible set to 0.1 and set the maximum number of credible sets in a region to three. We defined the lead variant of the credible set as the variant with the maximum posterior inclusion probability. To match a signal to credible set, we required that the lead variant be in the credible set.

<u>Genetic Correlation with Comorbid Traits</u>

We applied LD score regression (LDSC)[14,15] to estimate the genetic correlation between HS and celiac disease[16], IBD[17], polycystic ovarian syndrome[18], rheumatoid arthritis[19], type 2 diabetes[20], atopic dermatitis[21], psoriasis[22], schizophrenia[23], and asthma[24]. We

applied a Bonferroni correction to correct for multiple testing, setting a significance threshold of 0.0056 (α = 0.05/9 studies). We reviewed the lead HS variants in the GWAS Catalog[25] to identify reported associations with related traits.

Candidate Genes and Regulatory Elements

To prioritize potential candidate genes and identify variants within gene coding regions, we generated variant-level annotation using WGSA Annotator[26]. Next, we tested for evidence of colocalization between HS signals and signals from ten sources of expression quantitative trait loci (eQTL) data in HS-relevant tissues: skin (lower leg and subrapubic), lymphocytes, breast mammary tissue, and spleen from GTEx[27], two skin datasets from TwinsUK[28,29], blood from eQTLGen[30], and skin from the Psoriasis Treatment with Abatacept and Ustekinumab: A Study of Efficacy (PAUSE)[31]. If studies were reported mapped to hg38, we lifted the data to hg19 using the UCSC LiftOver tool[7]. We used PLINK[13] (1.90b3) to identify signals with LD $r^2 \geq 0.5$ between an HS lead variant and an eQTL lead variant for a gene within 500 kb (40K EUR UKB LD reference) and assessed colocalization using coloc[32] (5.1.0.1, default parameters). We considered signals to be colocalized if the posterior probability for a shared causal variant (PP4) was ≥ 0.7.

To further prioritize candidate genes, we queried if genes within 2 Mb of a lead variant were differentially expressed between cell types of the scalp[33] or differentially expressed in HS lesions versus unaffected skin of HS patients[34]. The scalp genomic data consisted of data from 22 cell clusters in five major cell classes: keratinocytes, T lymphocytes, myeloid cells, fibroblasts, and endothelial cells. We used scalp cell-type expression levels of genes near the HS GWAS signals to investigate plausible candidate genes[33]. We obtained $\log_2$ fold-change values from 22 cell clusters to examine the relative expression of all protein-coding genes within 2 Mb of any lead HS variant. We used ATAC-seq signal to compare regulatory element activity between 9 scalp cell types. To compute ATAC-seq signal per cell type, we quantified the number of Tn5 transpositions in each donor for each cell type, normalized this data by reads per million mapped reads, and summed normalized signal across the donors for a given cell type using a combination of BEDTools[35] and UCSCtools[36].

To investigate spatial chromatin interactions between lead GWAS variants and potential target genes, we used HUGIn[37] to examine Hi-C chromatin interaction data from mesenchymal cells[38]. We analyzed mesenchymal cells because inflammatory status of mesenchymal stem cells may play a role in HS pathogenesis[39].

To identify potential regulatory elements that may be altered by HS signals, we compared the HS-associated variants to regions identified using the assay of transposase-accessible chromatin (ATAC-seq) chromatin accessibility data in human scalp cell types[33]. We tested if any lead variants or their LD proxies (LD $r^2 \geq 0.9$, 40K EUR UKB) were located within the bounds of reported scalp ATAC-seq accessible regions, both for specific cell types and combined cell types.

We prioritized protein-coding genes within a somewhat strict 100 kb of a lead variant; nearest protein-coding genes for signals without a protein-coding gene within 100 kb; or genes within 1 Mb of the lead variant with external evidence of HS-relevance.

Transcriptional Reporter Assays

We performed transcriptional reporter assays in the spontaneously immortalized human epidermal HaCaT cell line (Cytion). We cultured HaCaT cells in DMEM-high glucose (Sigma) supplemented with 10% fetal bovine serum and maintained at 37°C in a humidified incubator with 5% CO_2. To test the allelic differences in transcriptional activity, we designed PCR primers (**Table S3**) to amplify DNA fragments spanning rs981625 (406 bp) or a 3-variant haplotype of rs17226067, rs17825774, and rs17825799 (375 bp). We generated PCR products for both alleles or haplotypes and cloned them into luciferase reporter vector pGL4.23 (Promega), which contains a minimal promoter, in forward and reverse orientations with respect to the genome. The day before transfection, we plated 110,000 HaCaT cells per well in 24-well plates and co-transfected three to five sequence-verified luciferase reporter constructs with phRL-TK Renilla reporter vector (Promega) in duplicate wells using lipofectamine 3000 (Life Technologies) following the manufacturer's protocol. Luciferase and Renilla activity were measured after 48 hours of transfection using the dual-luciferase reporter assay system. We normalized luciferase to Renilla activity, calculated fold-change in luciferase activity relative to an empty vector and tested for differences in activity between alleles using two-tailed Student's t-tests. All experiments were repeated a second independent day and yielded comparable results.

References

1. Sun, Q. *et al.* Genetic Variants Associated With Hidradenitis Suppurativa. *JAMA Dermatol* **159**, 930–938 (2023).
2. Harris, K. M., Halpern, C. T., Smolen, A. & Haberstick, B. C. The National Longitudinal Study of Adolescent Health (Add Health) Twin Data. *Twin Research and Human Genetics* **9**, 988–997 (2006).
3. Harris, K. M. *et al.* Cohort Profile: The National Longitudinal Study of Adolescent to Adult Health (Add Health). *International Journal of Epidemiology* **48**, 1415–1415k (2019).
4. Taliun, D. *et al.* Sequencing of 53,831 diverse genomes from the NHLBI TOPMed Program. *Nature* **590**, 290–299 (2021).
5. Fuchsberger, C., Abecasis, G. R. & Hinds, D. A. minimac2: faster genotype imputation. *Bioinformatics* **31**, 782–784 (2015).
6. Das, S. *et al.* Next-generation genotype imputation service and methods. *Nat Genet* **48**, 1284–1287 (2016).
7. Hinrichs, A. S. *et al.* The UCSC Genome Browser Database: update 2006. *Nucleic Acids Res* **34**, D590-598 (2006).
8. METAL: fast and efficient meta-analysis of genomewide association scans | Bioinformatics | Oxford Academic. https://academic.oup.com/bioinformatics/article/26/17/2190/198154.
9. Yang, J., Lee, S. H., Goddard, M. E. & Visscher, P. M. GCTA: a tool for genome-wide complex trait analysis. *Am J Hum Genet* **88**, 76–82 (2011).
10. Yang, J. *et al.* Conditional and joint multiple-SNP analysis of GWAS summary statistics identifies additional variants influencing complex traits. *Nat Genet* **44**, 369–375, S1-3 (2012).
11. Perez, G. *et al.* The UCSC Genome Browser database: 2025 update. *Nucleic Acids Res* **53**, D1243–D1249 (2025).
12. Zou, Y., Carbonetto, P., Wang, G. & Stephens, M. Fine-mapping from summary data with the “Sum of Single Effects” model. *PLOS Genetics* **18**, e1010299 (2022).
13. Purcell, S. *et al.* PLINK: a tool set for whole-genome association and population-based linkage analyses. *Am J Hum Genet* **81**, 559–575 (2007).
14. Bulik-Sullivan, B. *et al.* An atlas of genetic correlations across human diseases and traits. *Nat Genet* **47**, 1236–1241 (2015).
15. Bulik-Sullivan, B. K. *et al.* LD Score regression distinguishes confounding from polygenicity in genome-wide association studies. *Nat Genet* **47**, 291–295 (2015).
16. Jiang, L., Zheng, Z., Fang, H. & Yang, J. A generalized linear mixed model association tool for biobank-scale data. *Nat Genet* **53**, 1616–1621 (2021).
17. Liu, J. Z. *et al.* Association analyses identify 38 susceptibility loci for inflammatory bowel disease and highlight shared genetic risk across populations. *Nat Genet* **47**, 979–986 (2015).
18. Day, F. *et al.* Large-scale genome-wide meta-analysis of polycystic ovary syndrome suggests shared genetic architecture for different diagnosis criteria. *PLoS Genet* **14**, e1007813 (2018).
19. Saevarsdottir, S. *et al.* Multiomics analysis of rheumatoid arthritis yields sequence variants that have large effects on risk of the seropositive subset. *Ann Rheum Dis* **81**, 1085–1095 (2022).

20. Suzuki, K. *et al.* Genetic drivers of heterogeneity in type 2 diabetes pathophysiology. *Nature* **627**, 347–357 (2024).
21. Budu-Aggrey, A. *et al.* European and multi-ancestry genome-wide association meta-analysis of atopic dermatitis highlights importance of systemic immune regulation. *Nat Commun* **14**, 6172 (2023).
22. Sakaue, S. *et al.* A cross-population atlas of genetic associations for 220 human phenotypes. *Nat Genet* **53**, 1415–1424 (2021).
23. Trubetskoy, V. *et al.* Mapping genomic loci implicates genes and synaptic biology in schizophrenia. *Nature* **604**, 502–508 (2022).
24. Tsuo, K. *et al.* Multi-ancestry meta-analysis of asthma identifies novel associations and highlights the value of increased power and diversity. *Cell Genom* **2**, 100212 (2022).
25. Cerezo, M. *et al.* The NHGRI-EBI GWAS Catalog: standards for reusability, sustainability and diversity. *Nucleic Acids Research* **53**, D998–D1005 (2025).
26. Liu, X. *et al.* WGSA: an annotation pipeline for human genome sequencing studies. *J Med Genet* **53**, 111–112 (2016).
27. GTEx Consortium. The GTEx Consortium atlas of genetic regulatory effects across human tissues. *Science* **369**, 1318–1330 (2020).
28. Shore, C. J. *et al.* Genetic effects on the skin methylome in healthy older twins. *Am J Hum Genet* **111**, 1932–1952 (2024).
29. Kerimov, N. *et al.* A compendium of uniformly processed human gene expression and splicing quantitative trait loci. *Nat Genet* **53**, 1290–1299 (2021).
30. Võsa, U. *et al.* Large-scale cis- and trans-eQTL analyses identify thousands of genetic loci and polygenic scores that regulate blood gene expression. *Nat Genet* **53**, 1300–1310 (2021).
31. Xiao, Q. *et al.* Immunosuppression causes dynamic changes in expression QTLs in psoriatic skin. *Nat Commun* **14**, 6268 (2023).
32. Giambartolomei, C. *et al.* Bayesian test for colocalisation between pairs of genetic association studies using summary statistics. *PLoS Genet* **10**, e1004383 (2014).
33. Ober-Reynolds, B. *et al.* Integrated single-cell chromatin and transcriptomic analyses of human scalp identify gene-regulatory programs and critical cell types for hair and skin diseases. *Nat Genet* **55**, 1288–1300 (2023).
34. Freudenberg, J. M. *et al.* A Hidradenitis Suppurativa molecular disease signature derived from patient samples by high-throughput RNA sequencing and re-analysis of previously reported transcriptomic data sets. *PLoS One* **18**, e0284047 (2023).
35. Quinlan, A. R. & Hall, I. M. BEDTools: a flexible suite of utilities for comparing genomic features. *Bioinformatics* **26**, 841–842 (2010).
36. Kent, W. J. *et al.* The human genome browser at UCSC. *Genome Res* **12**, 996–1006 (2002).
37. Martin, J. S. *et al.* HUGIn: Hi-C Unifying Genomic Interrogator. *Bioinformatics* **33**, 3793–3795 (2017).
38. Schmitt, A. D. *et al.* A Compendium of Chromatin Contact Maps Reveals Spatially Active Regions in the Human Genome. *Cell Rep* **17**, 2042–2059 (2016).
39. Campanati, A. *et al.* Pathogenetic Characteristics of Mesenchymal Stem Cells in Hidradenitis Suppurativa. *JAMA Dermatol* **154**, 1184–1190 (2018).
