# Supplementary material for: Genomic loci and molecular genetic mechanisms for hidradenitis suppurativa
Source: Br J Dermatol. 2025 Jul 12;193(5):948–58. doi: 10.1093/bjd/ljaf277 (PMC12279254; doi:10.1093/bjd/ljaf277)
Supplement: ljaf277_Supplementary_Data [file ljaf277_supplementary_data.zip › HS meta-analysis Supplementary Figures 20250522.pdf]

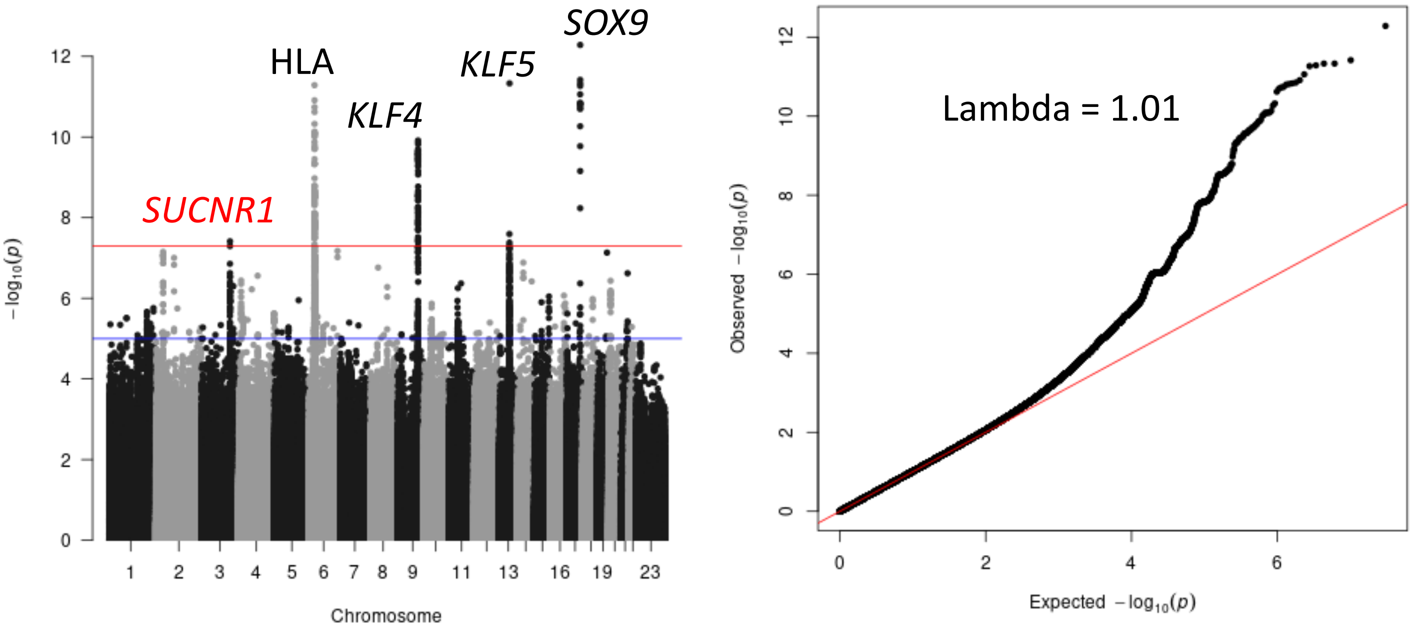

Supplementary Figure S1: Quantile-quantile and Manhattan plots of the associations between variants and HS in the GWAS analysis of all participants. Previously reported loci are in black; the new locus (P-value < 5×10^{-8}) is colored in red.

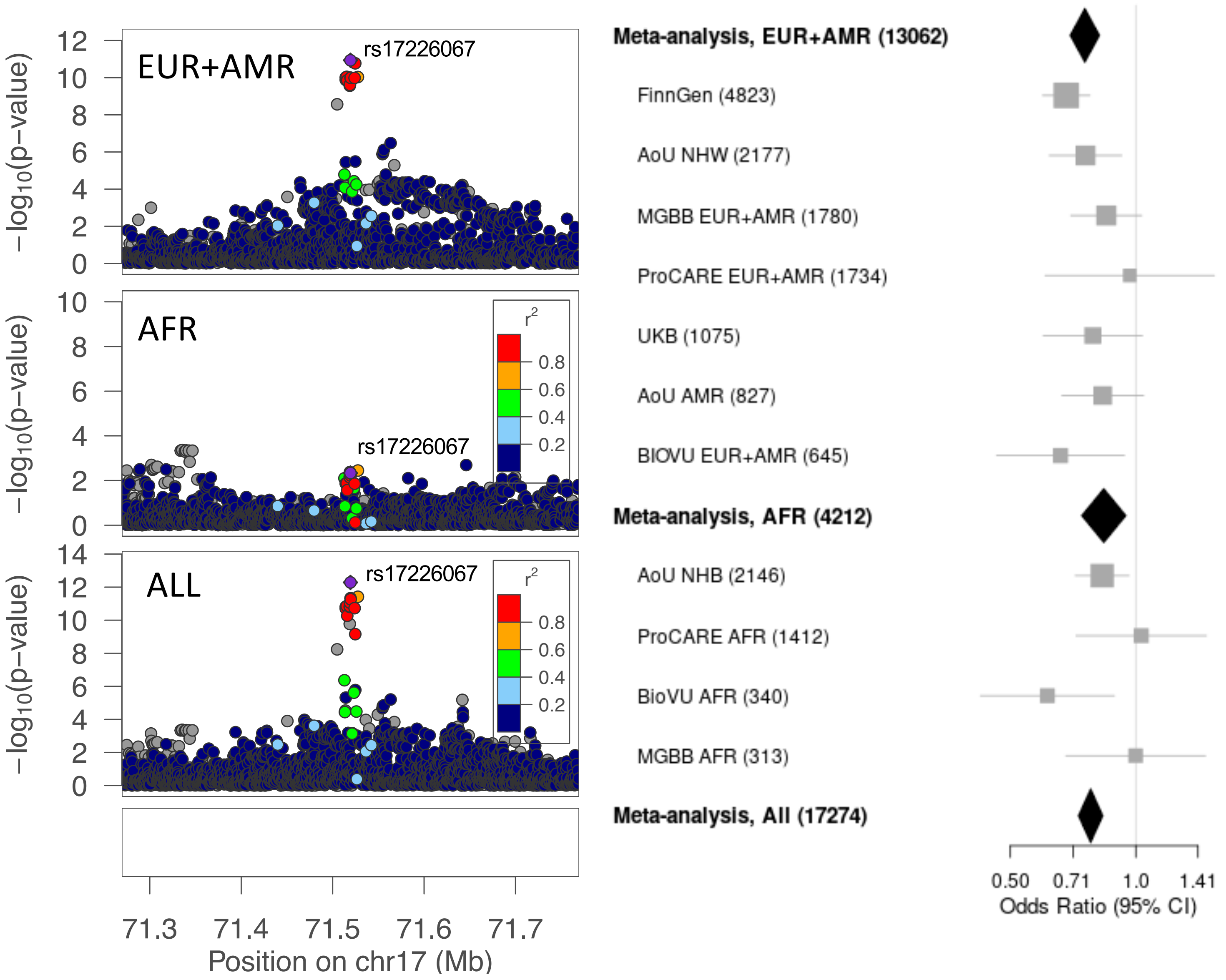

Supplementary Figure S2: *SOX9* HS locus. Left: Variant association with HS in EUR+AMR (top), AFR (center), and all-population (bottom) meta-analyses. **Right:** Forest plot of GWAS results for lead variant rs17226067. Numbers in parentheses denote effective sample sizes.

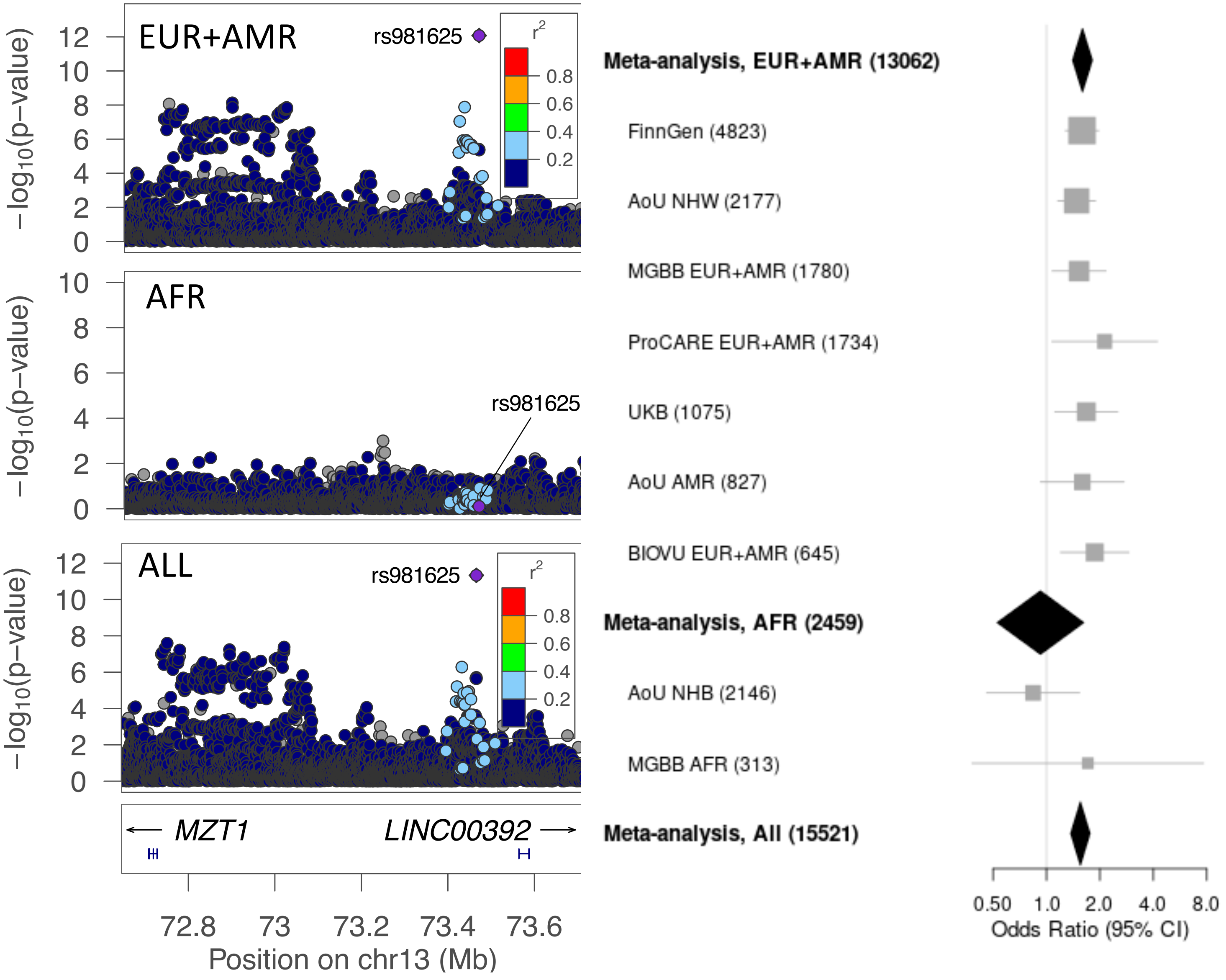

Supplementary Figure S3: *KLF5* HS locus, 1st signal. Left: Variant association with HS in EUR+AMR (top), AFR (center), and all-population (bottom) meta-analyses. **Right:** Forest plot of GWAS results for 1st signal lead variant rs981625 (shown in red on left plots). Numbers in parentheses denote effective sample sizes. rs981625 is more common in EUR+AMR (MAF = 0.06) than AFR (MAF = 0.01); only two AFR studies had rs981625 MAF ≥ 1% so sample size is low.

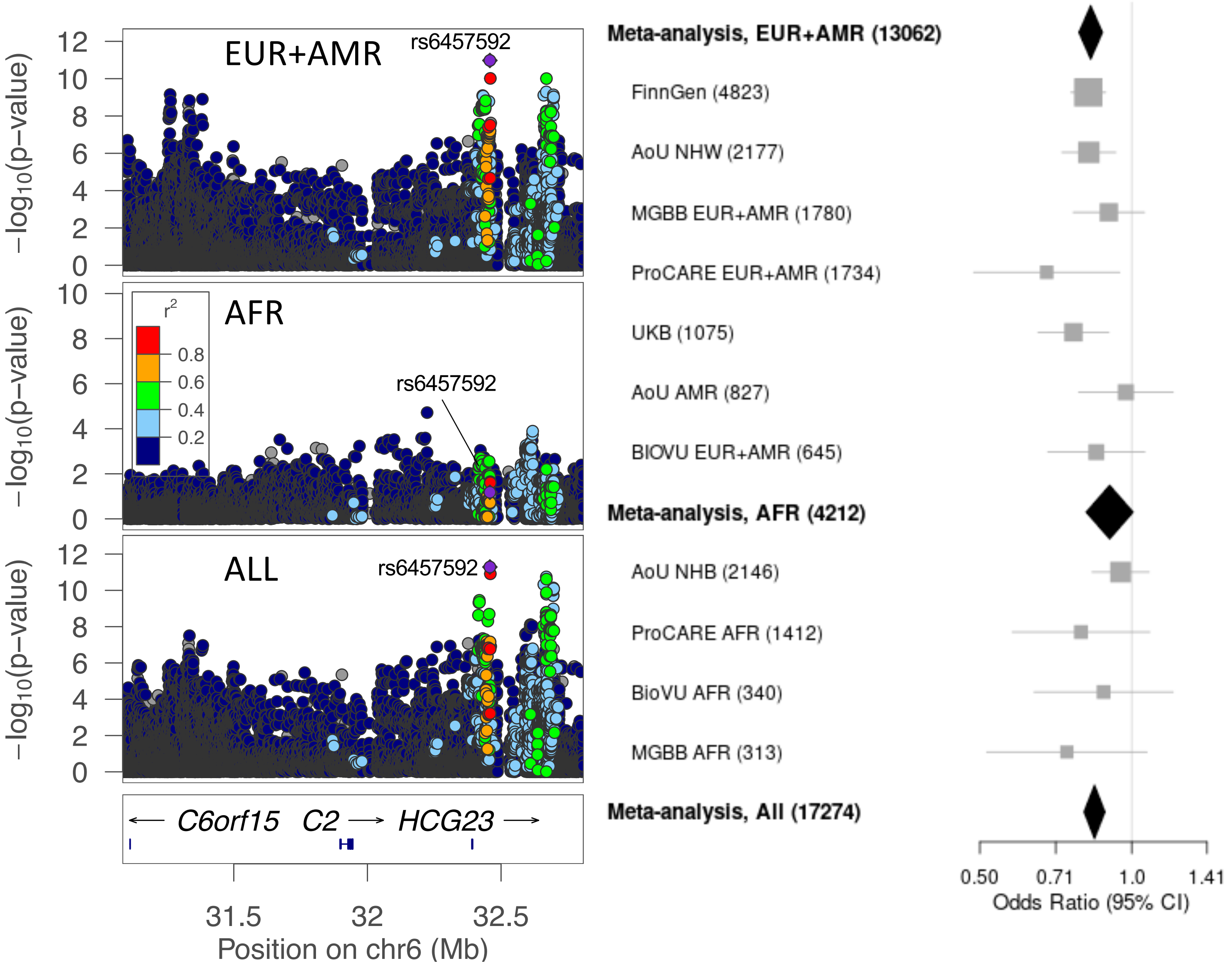

Supplementary Figure S4: *HLA* HS locus, 1st signal. Left: Variant association with HS in EUR+AMR (top), AFR (center), and all-population (bottom) meta-analyses. **Right:** Forest plot of GWAS results for 1st signal lead variant rs6457592. Numbers in parentheses denote effective sample sizes.

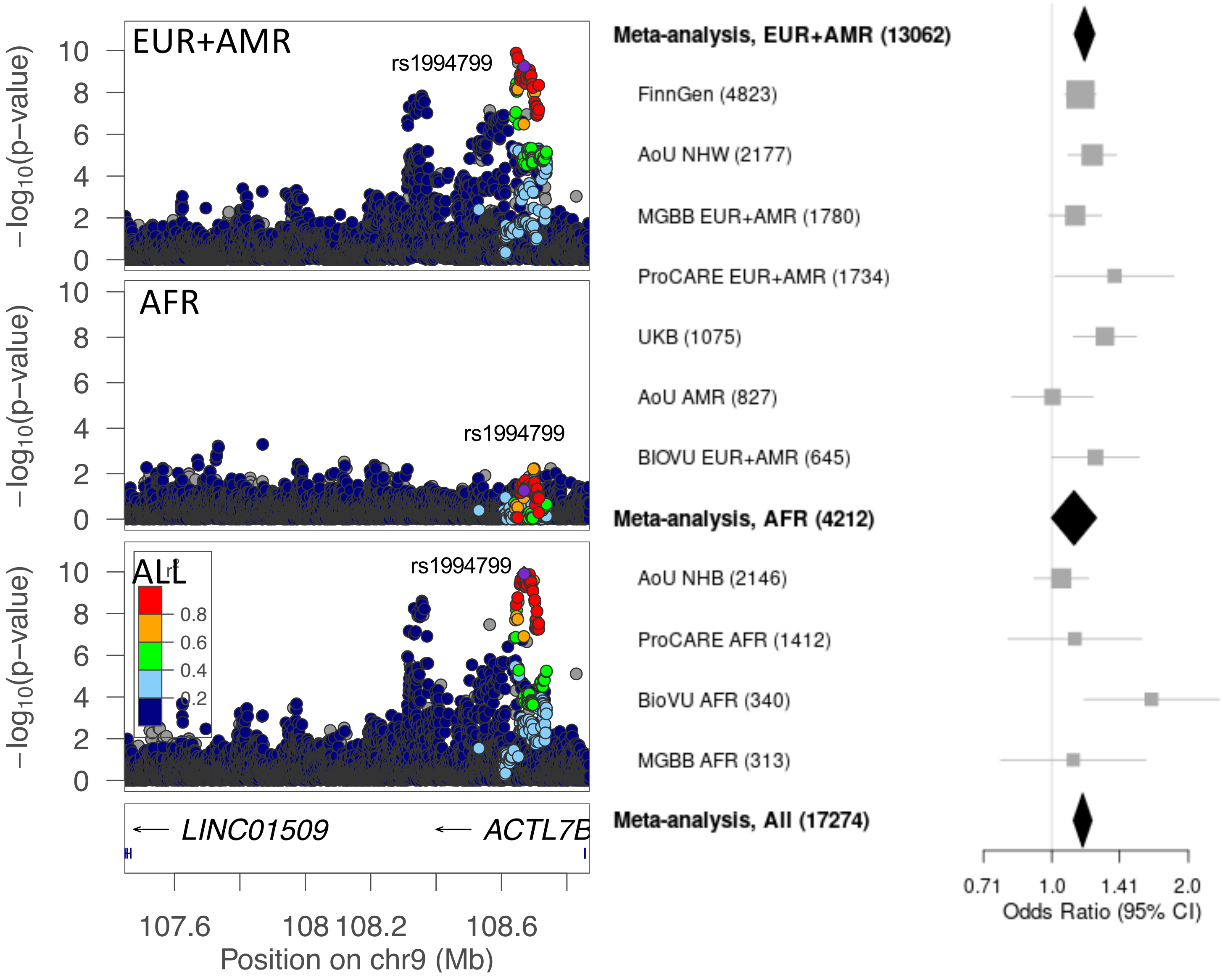

Supplementary Figure S5: *KLF4* HS locus, 1st signal. Left: Variant association with HS in EUR+AMR (top), AFR (center), and all-population (bottom) meta-analyses. **Right:** Forest plot of GWAS results for 1st signal lead variant rs1994799. Numbers in parentheses denote effective sample sizes.

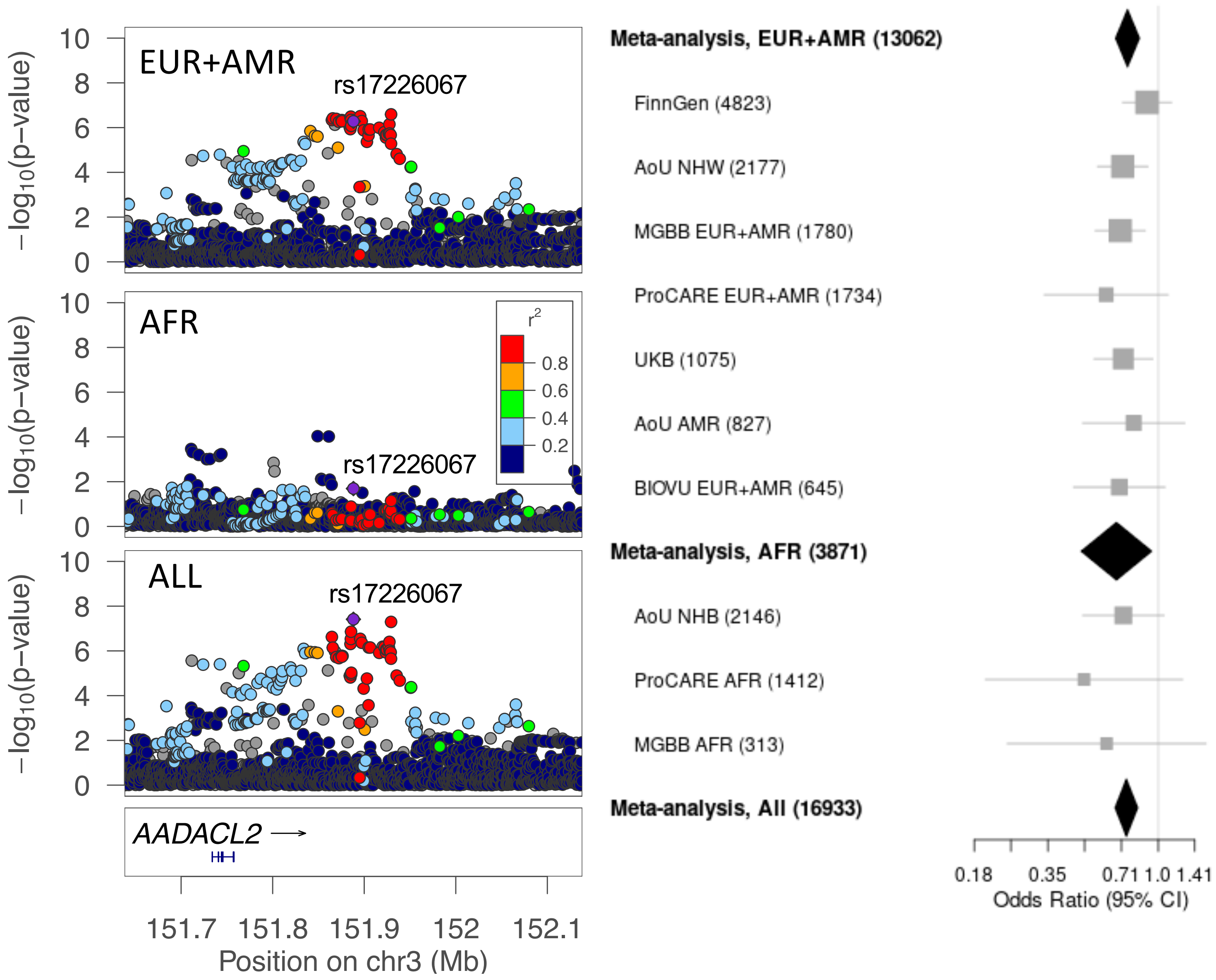

Supplementary Figure S6: *SUCNR1* HS locus. Left: Variant association with HS in EUR+AMR (top), AFR (center), and all-population (bottom) meta-analyses. **Right:** Forest plot of GWAS results for lead variant rs17226067. Numbers in parentheses denote effective sample sizes.

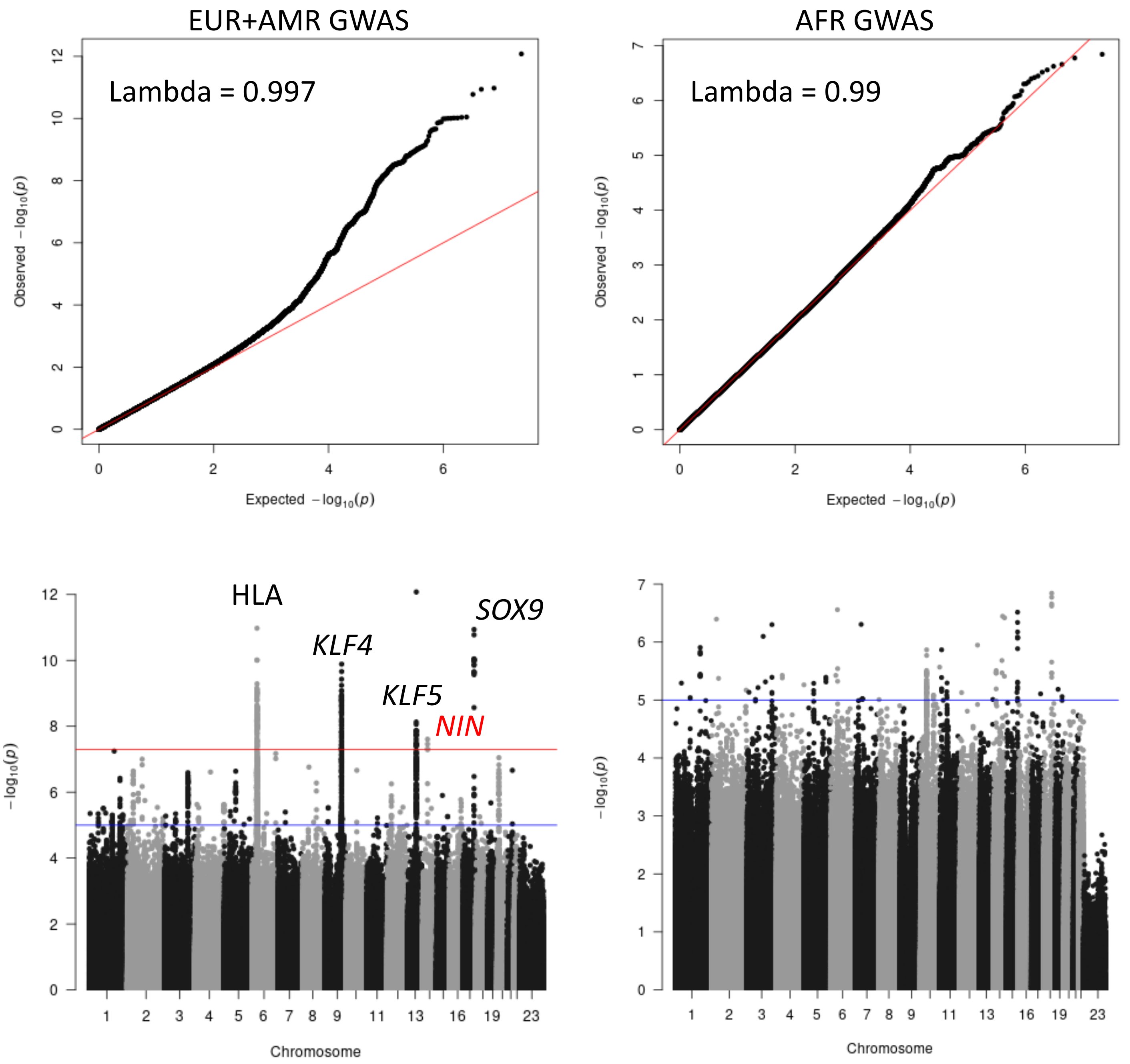

Supplementary Figure S7: Quantile-quantile and Manhattan plots of the associations between variants and HS in EUR+AMR (left) and AFR (right) GWAS analyses. Loci in the all-ancestry results are in black; the new locus (P-value <5x10^{-8}) is colored in red.

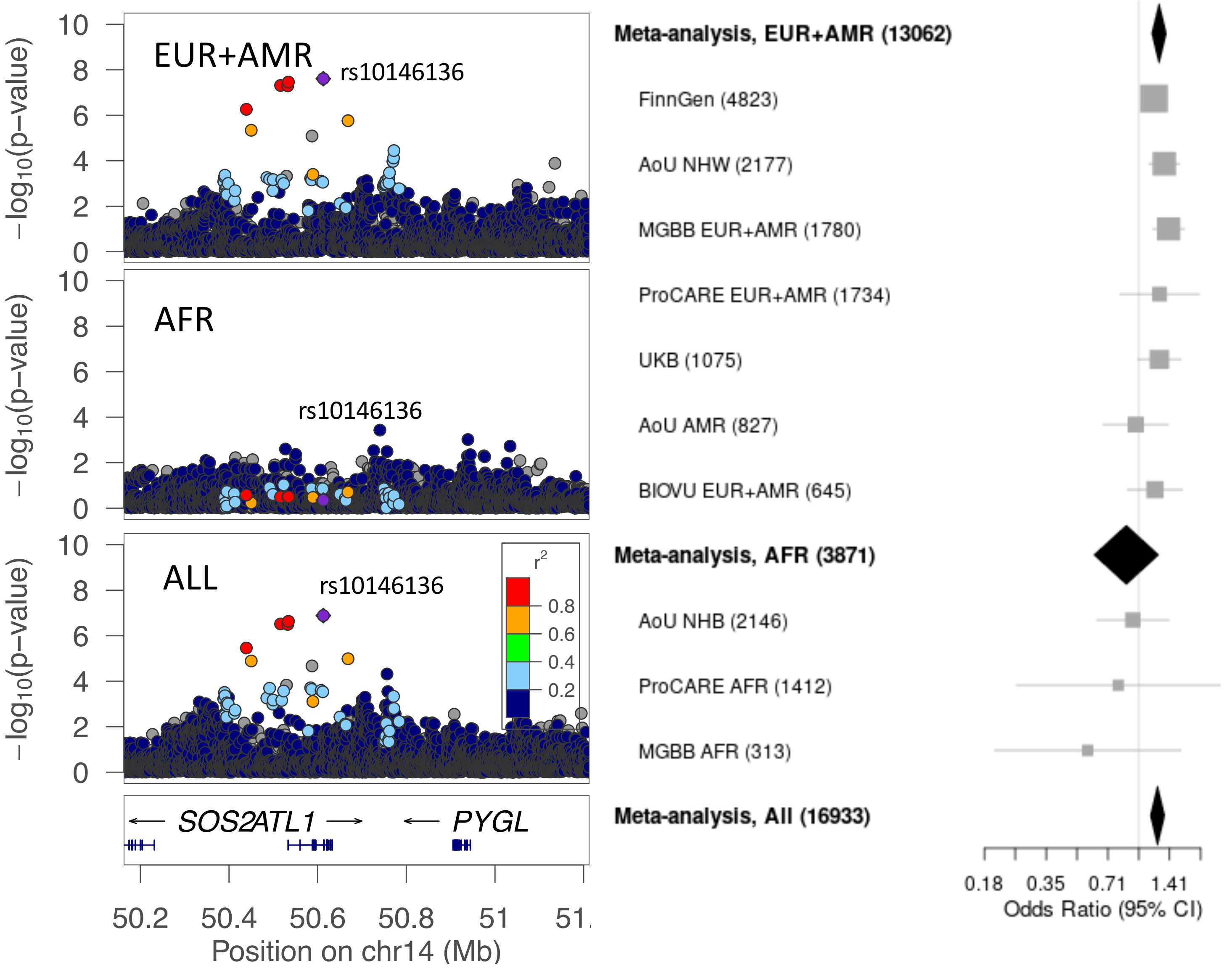

Supplementary Figure S8: Variant association with HS at the locus near *NIN*. Left: Variant association with HS in EUR+AMR (top), AFR (center), and all-population (bottom) meta-analyses. The plot for EUR+AMR HS meta-analysis (**top**) versus the all-ancestry HS meta-analysis (**bottom**) shows that while the all-ancestry results narrowly missed genome-wide significance (P-value = 1.3×10^{-7}), the signal is similar. **Right:** Forest plot of GWAS results for *NIN* lead variant rs10146136. Numbers in parentheses denote effective sample sizes. The lead variant is common in European populations (1000G EUR MAF = 13.3%) but rare in African populations (1000G AFR MAF = 0.5%), leading to uncertainty in effect in AFR studies.

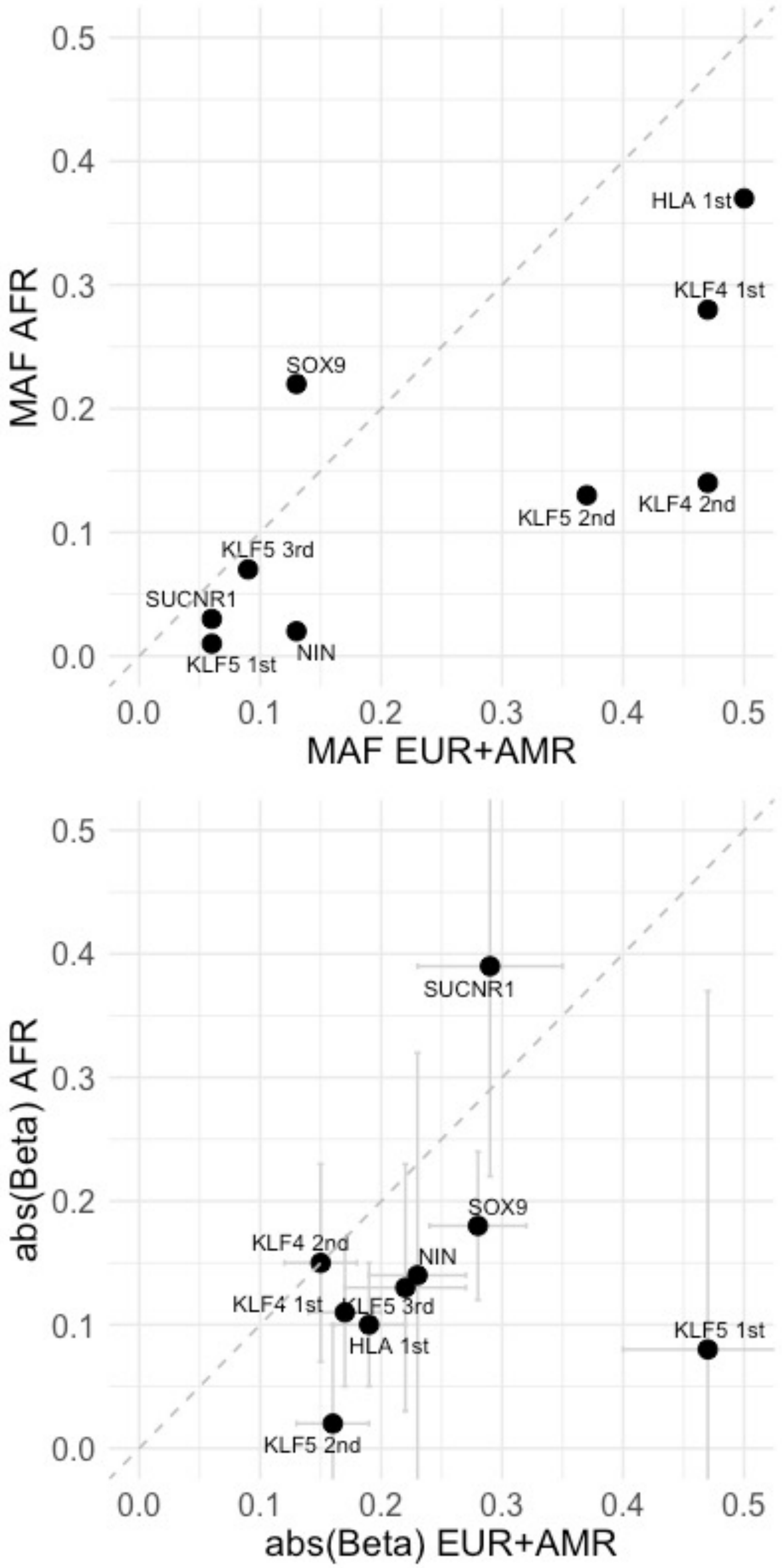

Supplementary Figure S9: Allele frequency and beta size differences for sex-combined lead variants from EUR+AMR and AFR meta-analyses. The second HLA signal was excluded from the AFR meta-analysis because it was only represented in one AFR cohort. **Top:** For all but one variant (SOX9), MAF was lower in AFR than EUR+AMR. **Bottom**: Effect sizes (absolute value of the betas) also tend to be smaller in AFR versus EUR+AMR.

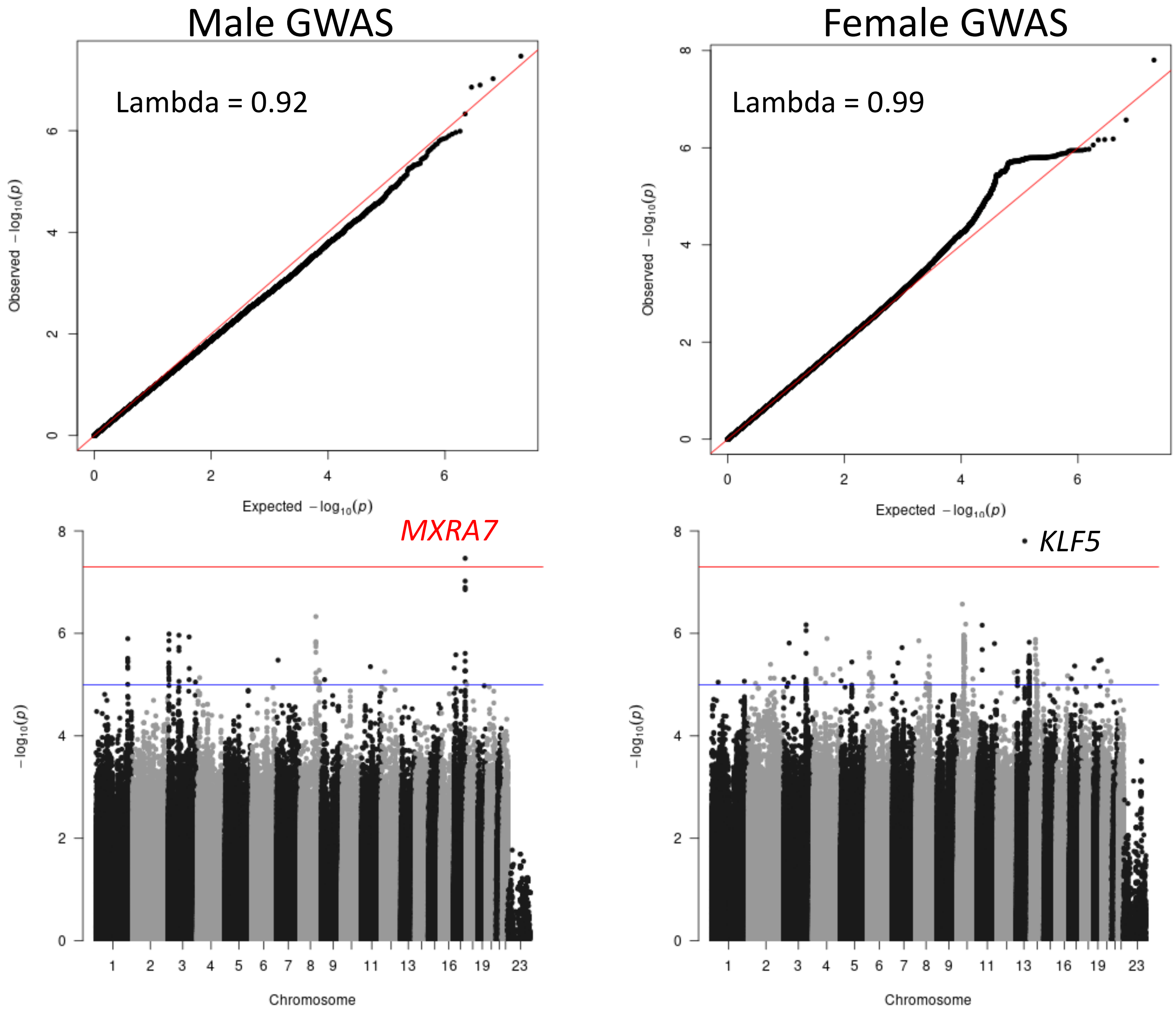

Supplementary Figure S10: QQ and Manhattan plots of the associations between variants and HS in males (left) versus females (right). Loci in the all-ancestry results are in black; the new locus (P-value < $5x10^{-8}$) is colored in red. Despite an effective sample size less than ¼ that of the female-specific analysis, we found a new new locus in the male-only analysis. The only locus that reached genome-wide significance in the female-only analysis was the *KLF5* lead variant.

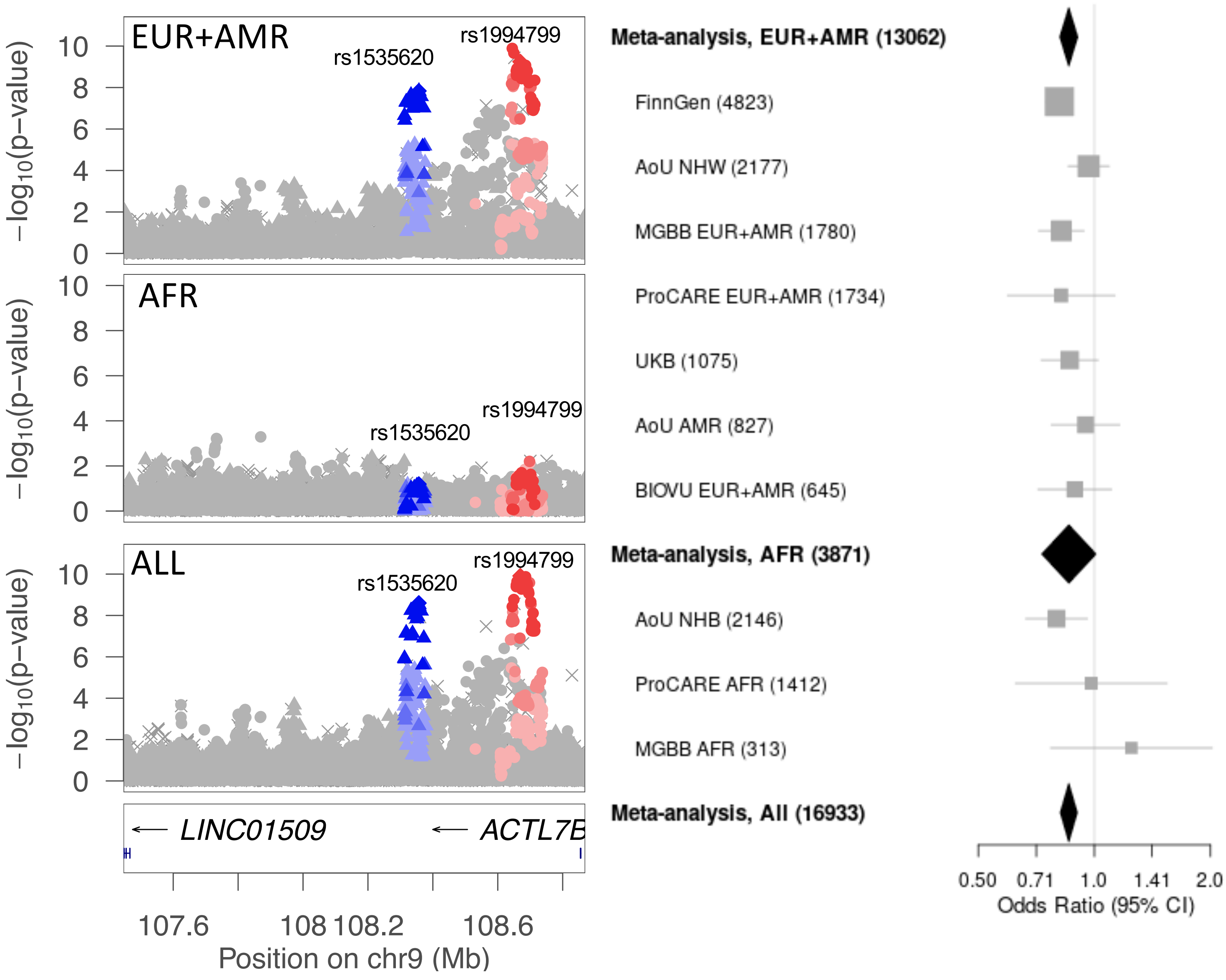

Supplementary Figure S11: Two conditionally distinct HS association signals within 1 Mb on chromosome 9. Left: GCTA identified a second HS association signal (shown in blue) in the EUR+AMR meta-analysis results near the primary *KLF4* signal (**top**), and in the all-population meta-analysis (**bottom**). No significant signals were detected in the AFR meta-analysis (**middle**). **Right:** Forest plot of GWAS results for 2nd lead variant rs1535620. Numbers in parentheses denote effective sample sizes. The lead variant is common in European populations (1000G EUR MAF = 49%) but less common in African populations (1000G AFR MAF = 7%).

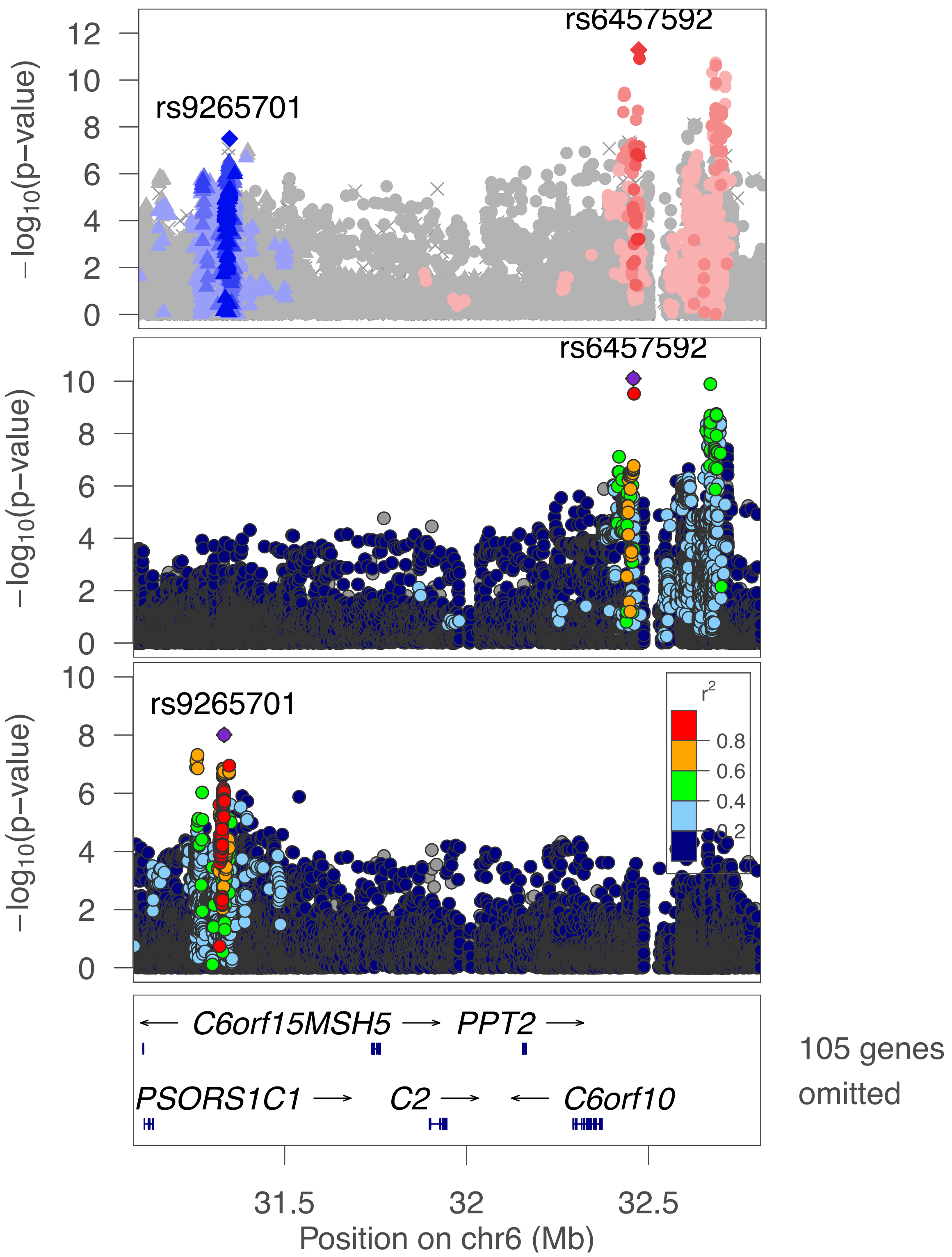

Supplementary Figure S12: Two HS association signals near HLA. Top: The primary HS signal (red) was in moderate LD with the previously-reported chr6 HS variant (previously reported lead rs3129859, LD r^2 = 0.35). The secondary HS signal is 1.1 Mb upstream. **Middle**: after conditioning on the second HS signal, the primary HS signal remains significant. **Bottom:** The secondary HS signal remains significant after conditioning on the primary HS signal.

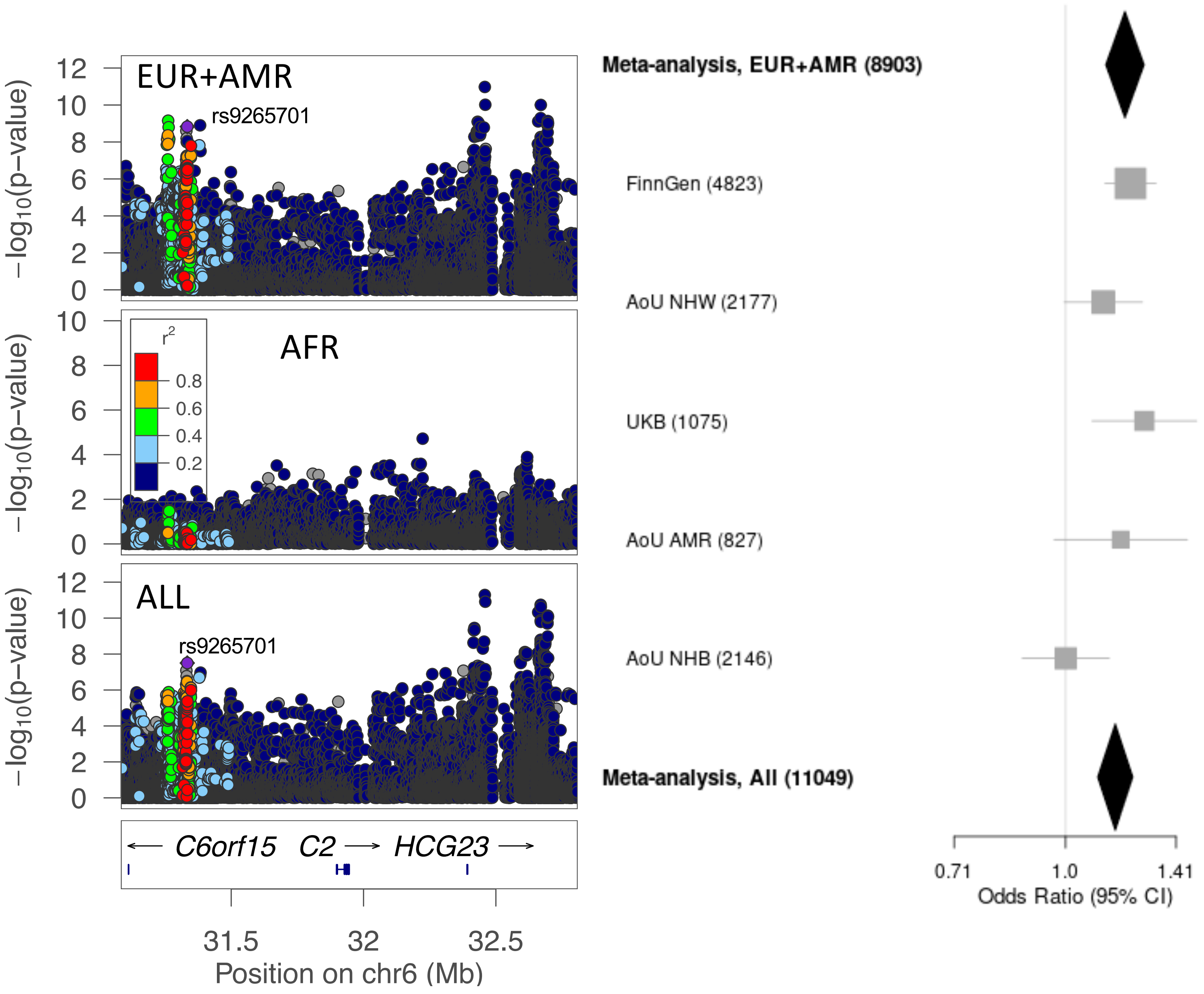

Supplementary Figure S13: *HLA* HS locus, 2nd signal. Left: Variant association with HS in EUR+AMR (top), AFR (center), and all-population (bottom) meta-analyses. The lead variant of the second signal was only represented at MAF ≥ 1% in one AFR study (All of Us AFR); the signal is not shown. **Right:** Forest plot of GWAS results for 1st signal lead variant rs9265701. Numbers in parentheses denote effective sample sizes. Since rs9265701 was only present in one AFR study (All of Us), the variant was not included in the meta-analysis.

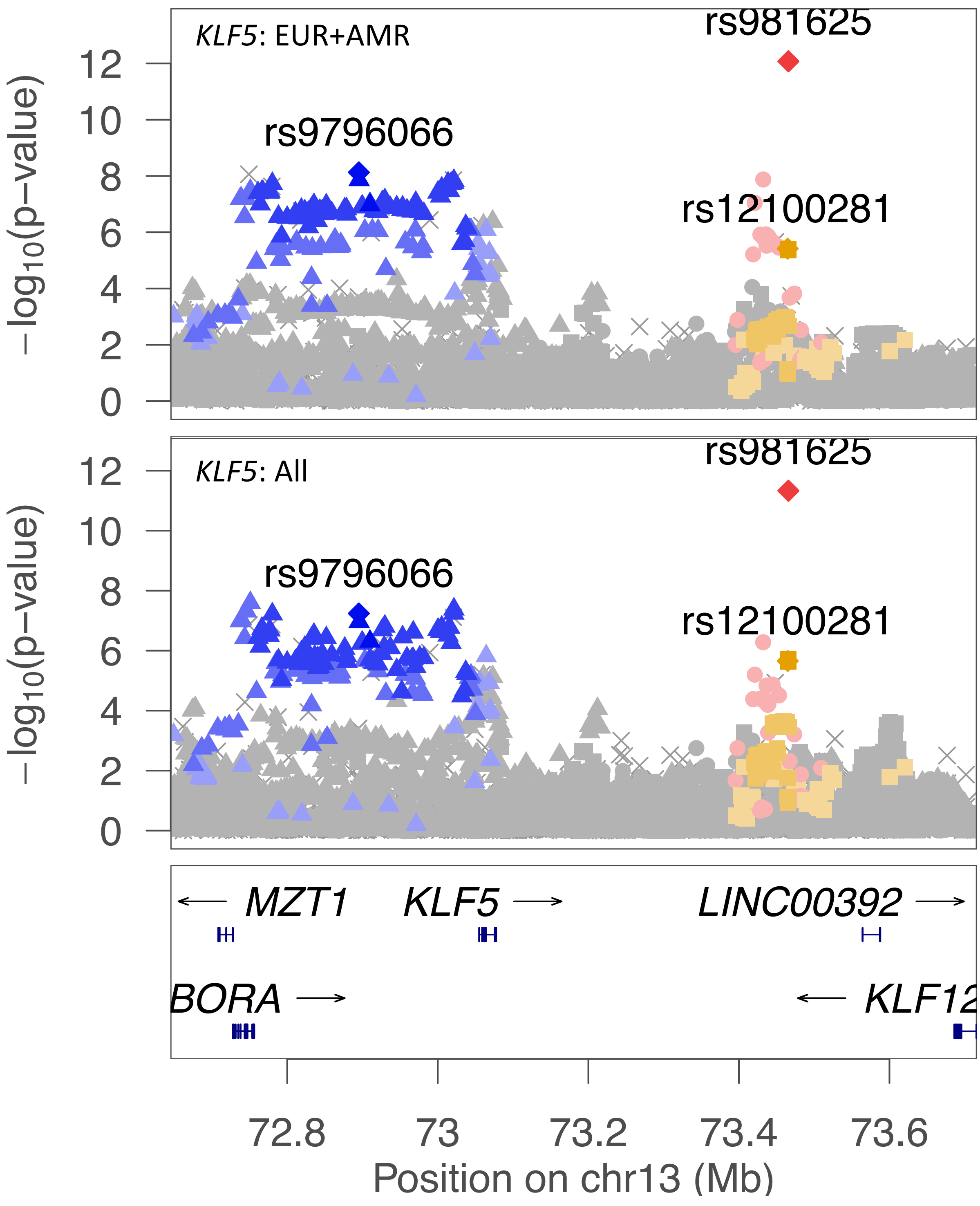

Supplementary Figure S14: Three HS association signals near *KLF5*. Top: GCTA identified a second and third HS signal in the EUR+AMR meta-analysis results (top) but did not identify multiple signals in the all-ancestry results. However, SuSiE identified credible sets in the all-ancestry HS GWAS meta-analysis results that corresponded to the three *KLF5* signals. The all-ancestry results are shown on bottom.

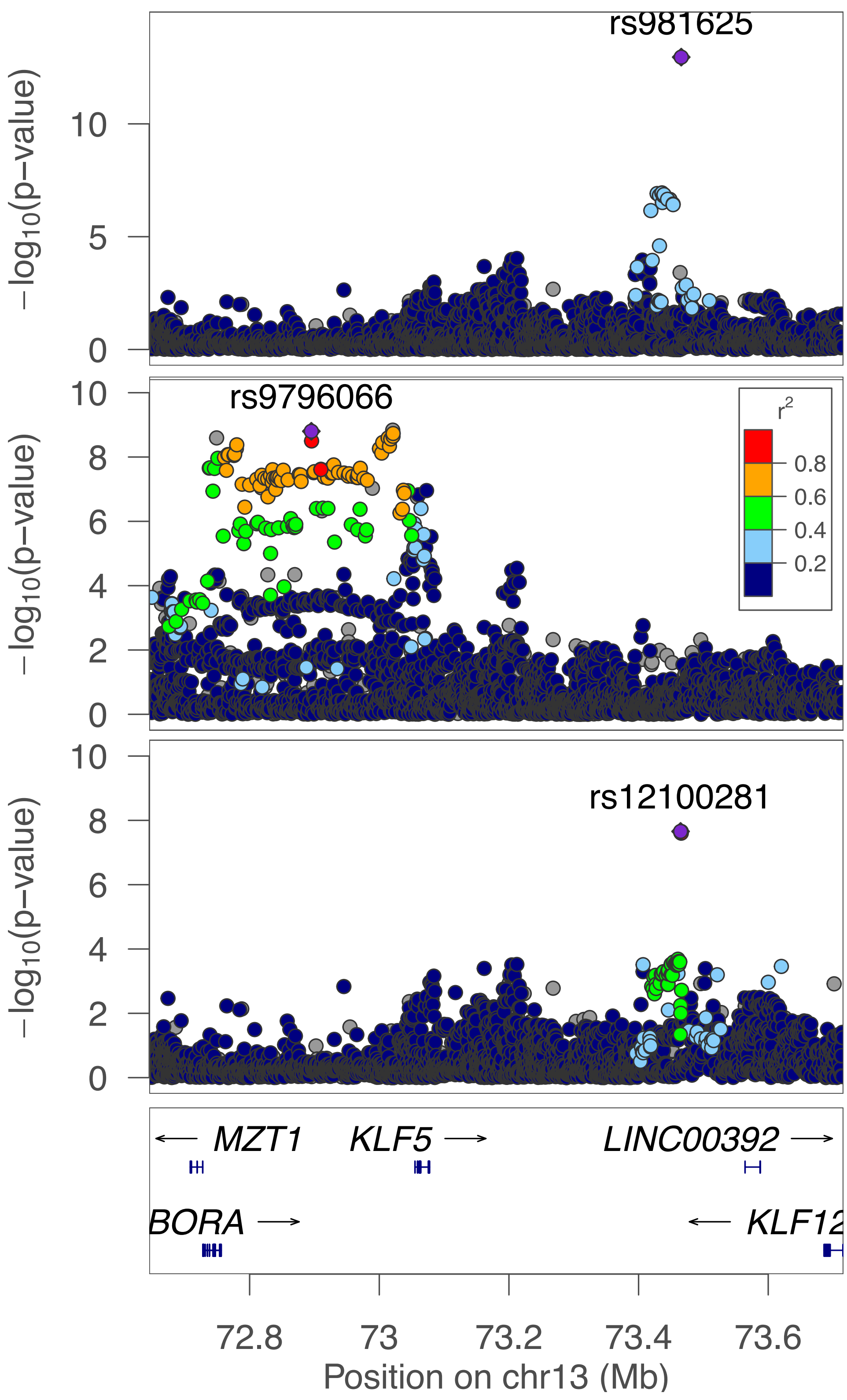

Supplementary Figure S15: Three HS association signals near *KLF5* in the EUR+AMR GWAS meta-analysis. The primary (**top**), secondary (**middle**), and tertiary (**bottom**) signals, after controlling for the effects of the other two HS association signals in the region.

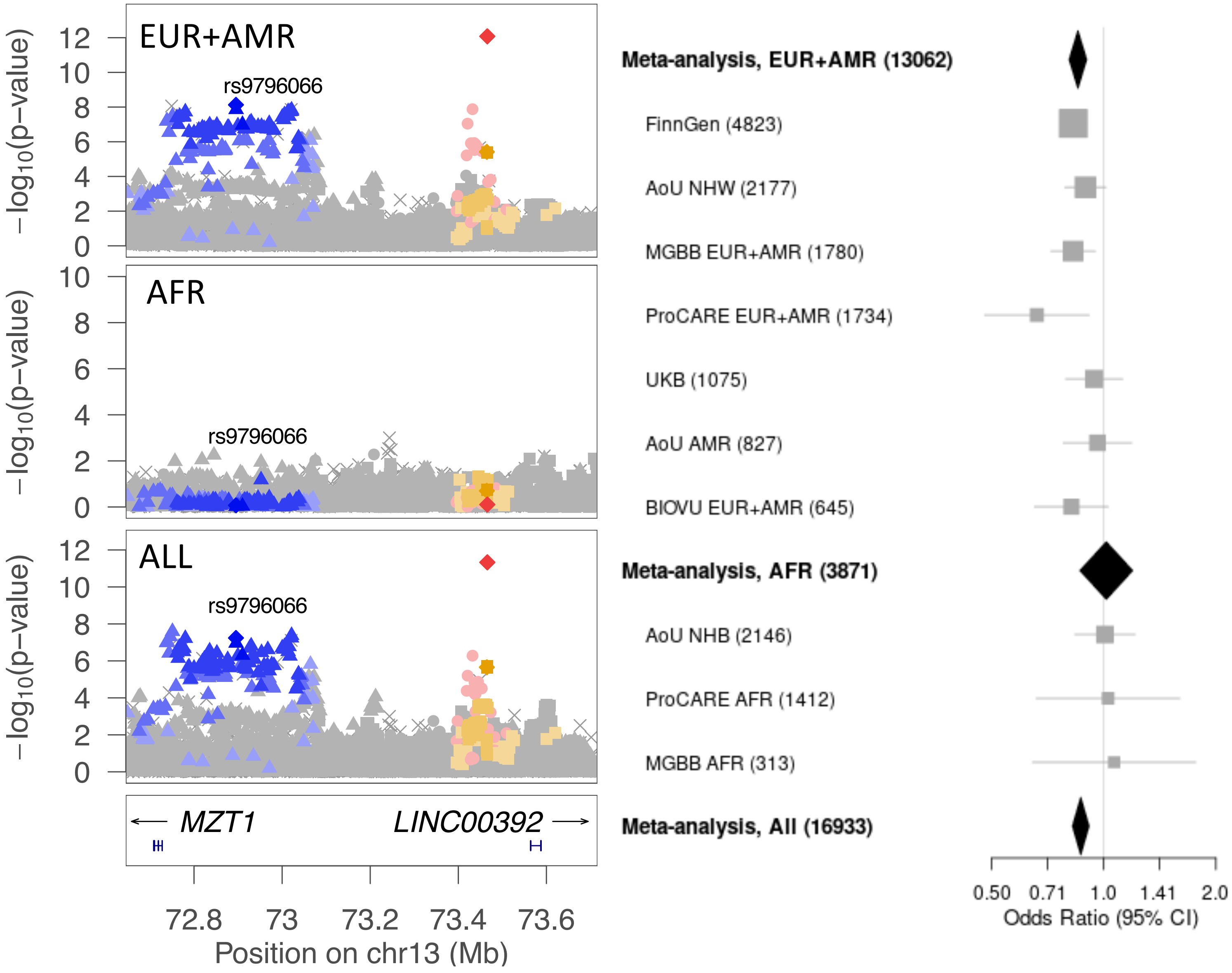

Supplementary Figure S16: *KLF5* HS locus, 2nd signal, shown in blue. Left: Variant association with HS in EUR+AMR (top), AFR (center), and all-population (bottom) meta-analyses. **Right:** Forest plot of GWAS results for 2nd signal lead variant rs9796066. Numbers in parentheses denote effective sample sizes.

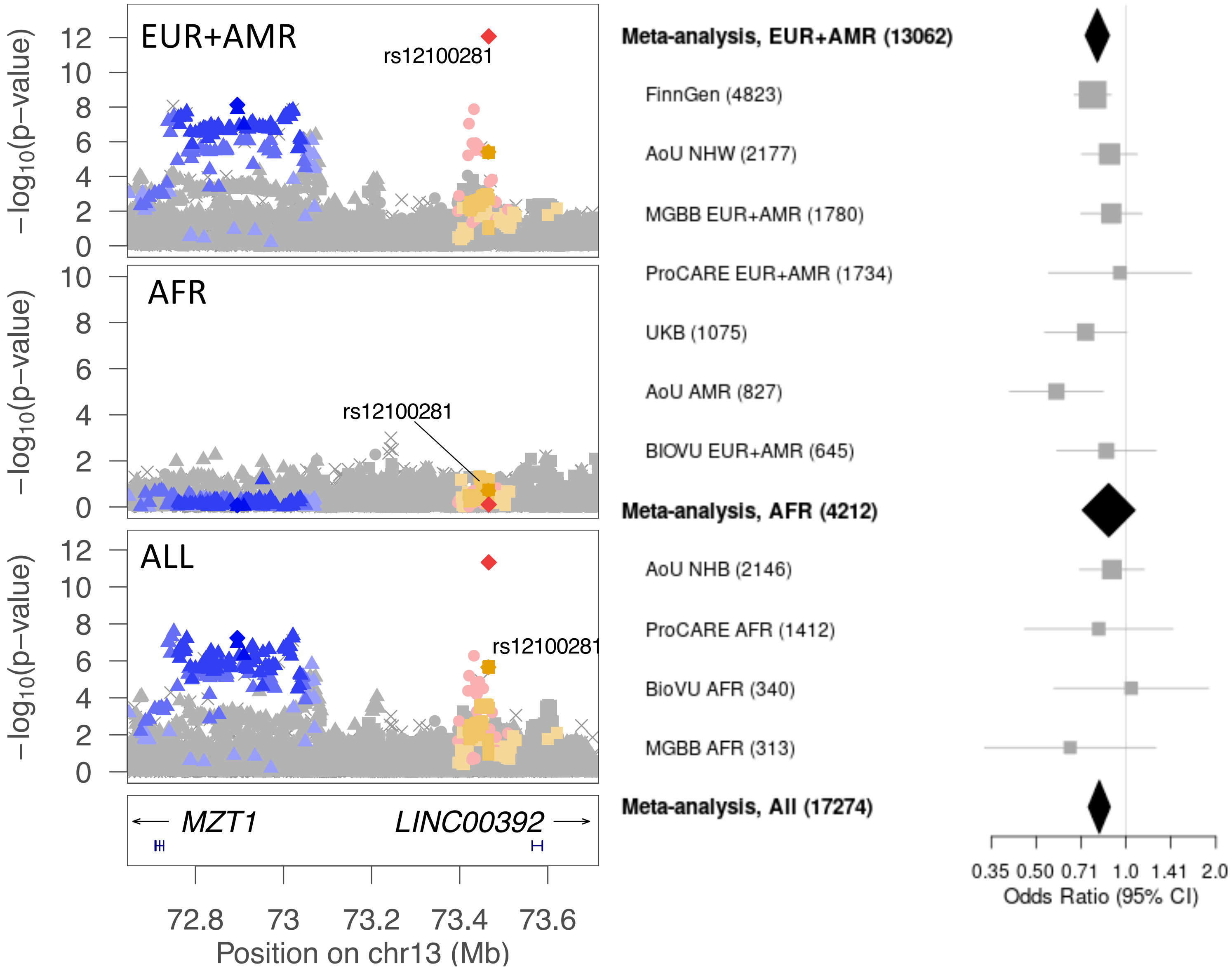

Supplementary Figure S17: *KLF5* HS locus, 3rd signal, shown in yellow. Left: Variant association with HS in EUR+AMR (top), AFR (center), and all-population (bottom) meta-analyses. **Right:** Forest plot of GWAS results for 2nd signal lead variant rs12100281. Numbers in parentheses denote effective sample sizes.

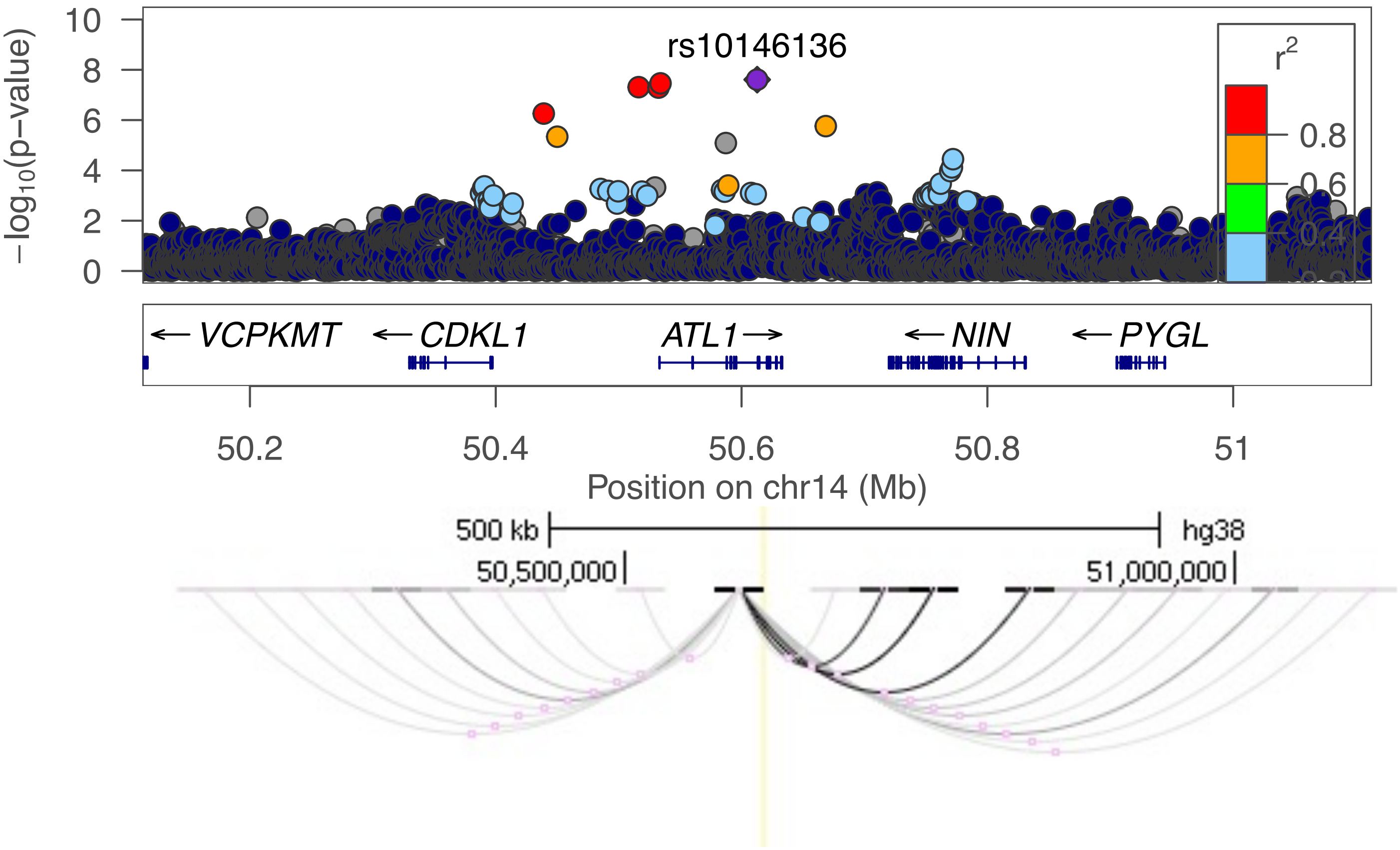

Supplementary Figure S18: Chromatin interactions between HS variants and the promoter region of *NIN*. Mesenchymal Hi-C data centered around the lead variant for the primary HS signal in this region (rs10146136) showed a chromatin interaction peak centered around *NIN*. **Top**: Variants associated with HS in the EUR+AMR GWAS meta-analysis. **Bottom**: Mesenchymal Hi-C data from Schmitt 2016 (PMID 27851967) plotted in the Genome Browser shows a chromatin interaction peak centered around *NIN*. The target regions immediately flanking the anchor region and target regions with FitHiC Q-values ≥ 0.01 are not shown. Arc colors are scaled by strength of association measured by $-\log_{10}$(P-value) with the darkest line showing the link with the strongest P-value.

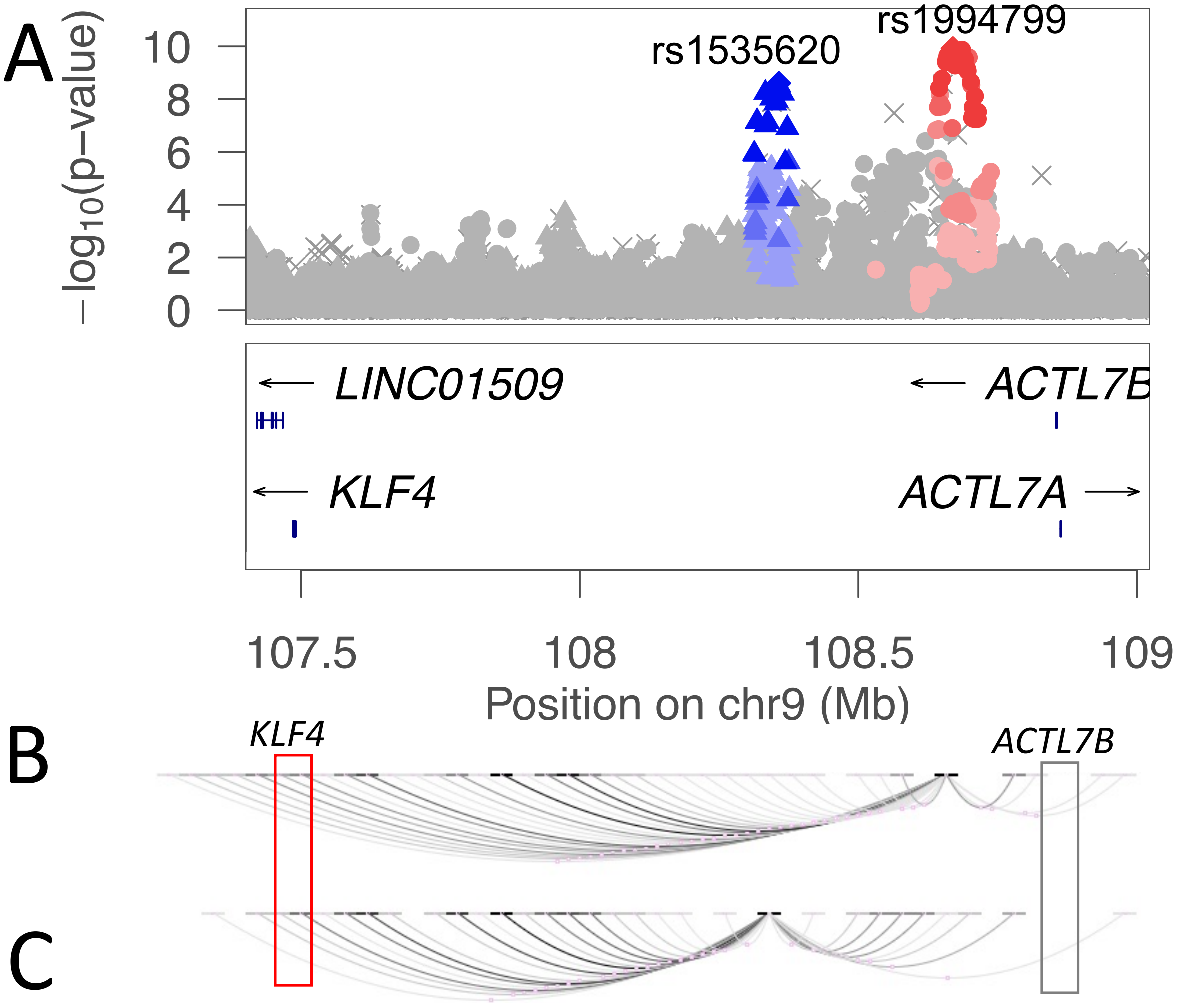

Supplementary Figure S19: Both conditionally distinct HS signals on chromosome 9 exhibited a significant Hi-C chromatin interaction peak in mesenchymal data. A: Variants associated with HS for two conditionally distinct signals. **B**: Mesenchymal Hi-C data anchored around the lead variant for the primary signal (rs1994799). **C**: Mesenchymal Hi-C data anchored around the lead variant for the secondary signal (rs1535620). The target regions immediately flanking the anchor regions and target regions with FitHiC Q-values ≥ 0.01 are not shown. Arc colors are scaled by strength of association measured by $-\log_{10}$(P-value) with the darkest lines showing the link with the strongest P-value. Hi-C data in the red boxes, centered around two prioritized genes *ACTL7B* and *KLF4*, are continued on the next image.

Mesenchymal Hi-C interaction: significant links at *KLF4*

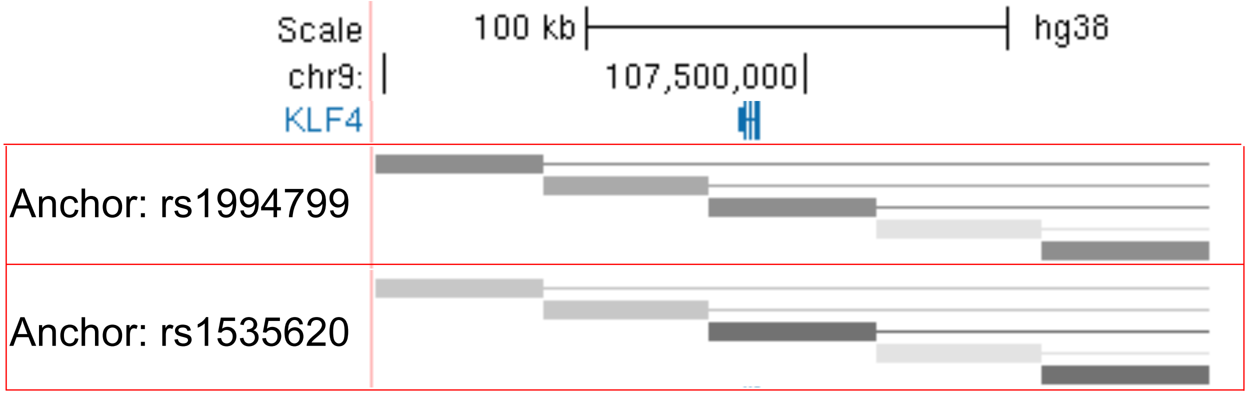

Mesenchymal Hi-C interaction: no significant links at *ACTL7B*

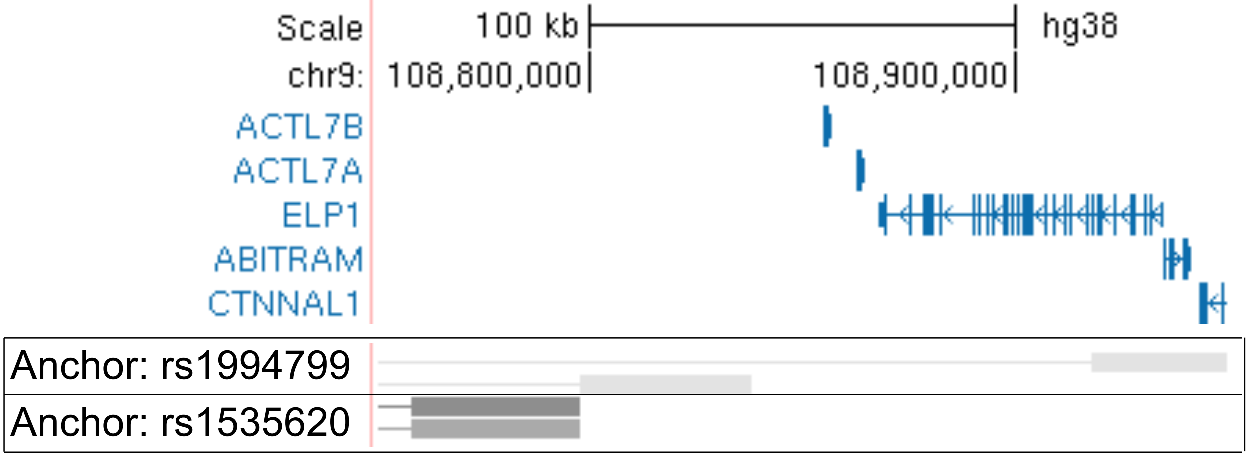

Supplementary Figure S19 Continued: Both conditionally distinct HS signals on chromosome 9 exhibited a significant Hi-C chromatin interaction peak in mesenchymal data. Top: Mesenchymal Hi-C data centered around *KLF4* with anchors at the primary and secondary lead variants (rs1994799 and rs1535620). Both lead variants on chromosome 9 exhibited a significant Hi-C chromatin interaction peak, linking them to the promoter region of *KLF4.* **Bottom***:* No significant links in mesenchymal Hi-C data centered around *ACTL7B*, with anchors at the primary and secondary lead variants for HS on chromosome 9. There is no evidence of a Hi-C chromatin interaction peak to link either lead variant on chromosome 9 with *ACTL7B* or *ACTL7A*.

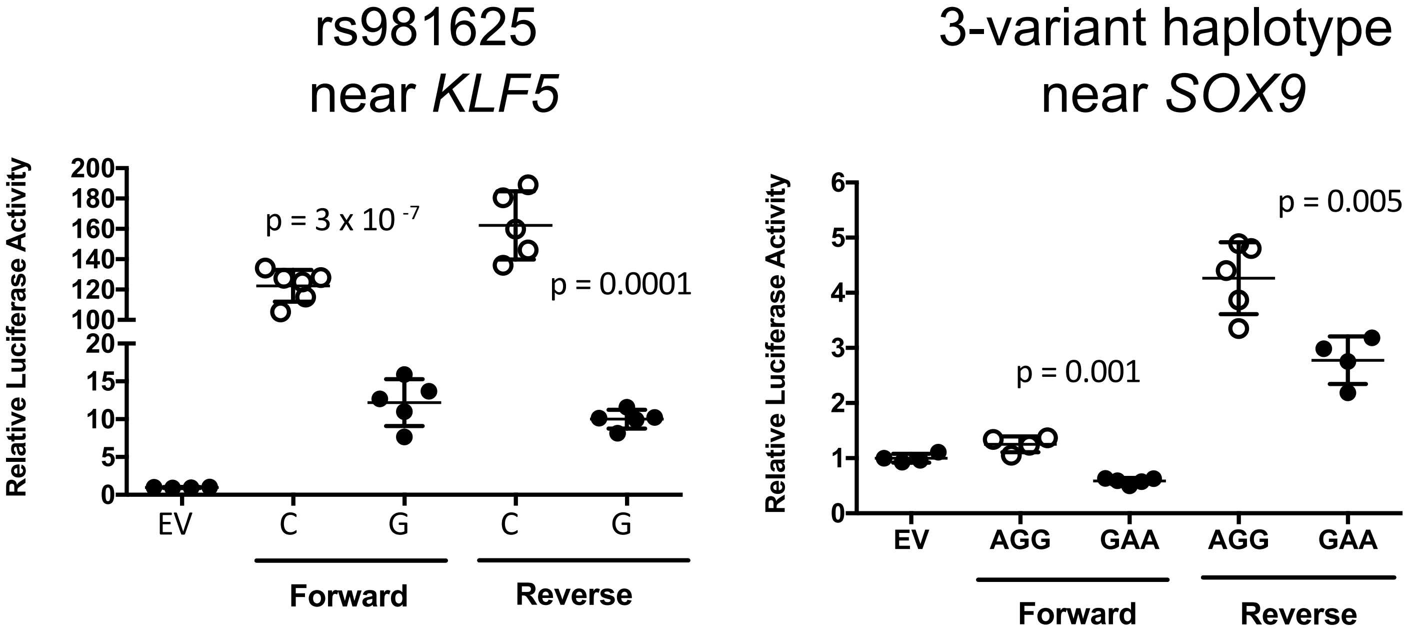

Supplementary Figure S20: Allelic differences in keratinocyte transcriptional activity for variants at the *KLF5* and *SOX9* loci. Left: At rs981625, the lead variant of the primary *KLF5* signal, the rs981625-C allele showed >10-fold greater transcriptional activity than the reference allele (P-value = 2.9 x 10^{-7}) and was associated with higher HS risk. **Right:** At a 3-variant haplotype including rs17226067-G, the lead *SOX9* variant, and its LD proxies, the haplotype including the rs17226067-G allele showed >2-fold reduced transcriptional activity versus the reference haplotype (P-value = 0.001).
